# Stochastic modelling of deep magmatic controls on porphyry copper deposit endowment

**DOI:** 10.1038/srep44523

**Published:** 2017-03-15

**Authors:** Massimo Chiaradia, Luca Caricchi

**Affiliations:** 1 Department of Earth Sciences, University of Geneva, Rue des Maraîchers 13, 1205, Geneva, Switzerland

## Abstract

Porphyry deposits, our main source of copper and of significant amounts of Mo, Re and Au, form at convergent margins in association with intermediate-felsic magmas. Although it is accepted that copper is transported and precipitated by fluids released by these magmas, the magmatic processes leading to the formation of economic deposits remain elusive. Here we perform Monte Carlo petrological and geochemical modelling to quantitatively link crustal magmatic processes and the geochemical signatures of magmas (i.e., Sr/Y) to the formation of porphyry Cu deposits of different sizes. Our analysis shows that economic deposits (particularly the largest ones) may only form in association with magma accumulated in the lower-middle crust (P > ~0.5 GPa) during ≥2–3 Ma, and subsequently transferred to and degassed in the upper crust over periods of up to ~2.0 Ma. Magma accumulation and evolution at shallower depths (<~0.4 GPa) dramatically reduces the potential of magmatic systems to produce economic deposits. Our modelling also predicts the association of the largest porphyry deposits with a specific Sr/Y interval (~100 ± 50) of the associated magmatic rocks, which is virtually identical to the range measured in giant porphyry copper deposits.

Porphyry deposits, suppliers of ~75% of the world’s copper and of significant proportions of Mo, Au, Re, are formed in subduction-related magmatic arcs^1^ and, to some extent, in post-subduction collisional zones^2^, where they are associated with magmas of intermediate to felsic compositions (mostly andesitic to dacitic) having calc-alkaline to high-K calc-alkaline affinity^1^. It is widely accepted that copper in porphyry-type deposits is transported and precipitated by fluids extracted from these magmas^3^,^4^,^5^. Therefore, the amount of copper that is ultimately precipitated in porphyry deposits is a function of the amount of magmatic fluids available and of the copper concentration in those fluids, assuming that precipitation efficiencies do not change significantly from one magmatic system to another. In turn, the mass of available fluids and their copper^6^ concentrations are directly related to the amount of magma and its volatile and copper content, because the transfer of copper from magma to fluid is modulated by a specific interval of fluid-melt partition coefficient values.

Geochronologic studies show that the total period of magmatic-hydrothermal activity ranges from a few tens of thousands of years up to almost 2 Ma in porphyry-type deposits of different sizes^7^,^8^,^9^,^10^,^11^,^12^ (Supplementary Information 1, Table S1.1). Such temporal constraints provide broad limits to the duration of magmatic-hydrothermal activity leading to the formation of these deposits. Additionally, although not fully understood, the relationship between magmas with high Sr/Y values (~100 ± 50) and porphyry formation has empirically proved positive in several major porphyry Cu deposits^13^,^14^,^15^ and has been increasingly used as a fertility indicator of magmatic systems during exploration.

All the above observations must be accounted for to develop quantitative models describing the genesis of porphyry-type deposits. Annen *et al*.^16^ have developed a thermal model to quantify the generation and evolution of intermediate to felsic magmas, i.e., those typically associated with porphyry deposits, which is valid for a broad range of physico-chemical conditions (P, T, H_2_O contents, magma fluxes) deemed appropriate for subduction-related magmatic systems. This thermal modeling shows that, for a given magma flux, the size of crustal magmatic systems, which ultimately controls the maximum amount of Cu that they can deliver, is controlled by the depth (pressure, P) and duration of magma accumulation (time, t).

Here, using a Monte Carlo approach, we combine the model of ref. 16 with models of H_2_O solubility in silicate melts^17^, petrological and geochemical modelling, as well as mass balance calculations, to quantify crustal depths and timescales of formation of magmatic systems best suited to produce economic porphyry Cu deposits of different sizes within the time intervals constrained by radiometric dating (Table S1.1 in Supplementary Information 1).

## Model Rationale

### Volumes of intermediate-felsic melts associated with porphyry Cu deposits

**Figure 1:**
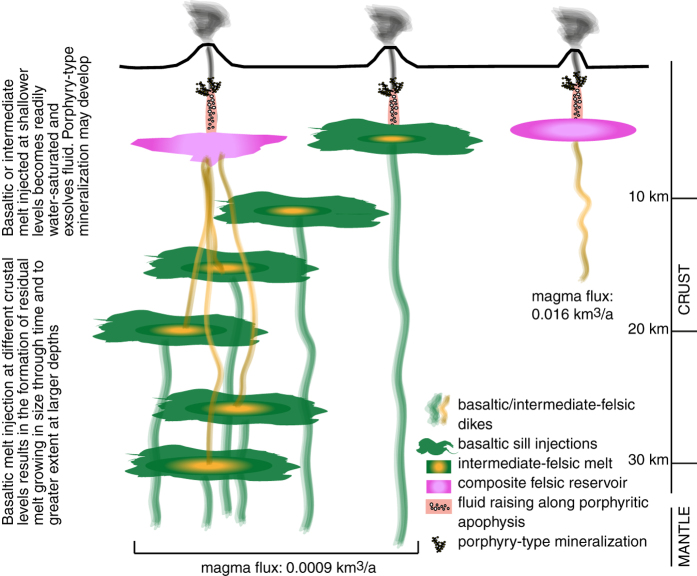
Conceptual model of intermediate-felsic melt generation in the arc crust (modified after ref. 16). In the arc environment intermediate/felsic melts form as a consequence of injection of basaltic sills at different crustal depths. They result from residual melts issued from the fractionation of the injected basalt that eventually mix with partial melts of the host rocks, forming a hybrid melt. Because of thermal constraints hybrid melts generated at greater depths can grow in size much more than hybrid melts formed at shallower depths after a given time since the onset of basaltic sill injection. Hybrid melts formed at deeper crustal levels are H_2_O-undersaturated and must rise to shallower crustal levels to be able to exsolve fluid and form porphyry deposits. Intermediate-felsic magmatic systems generated by basaltic melts injected at shallow crustal levels under average magma fluxes may exsolve fluid readily but are generally limited in volume. Intermediate-felsic magmas injected at shallower levels under high magma fluxes result in the transient construction of large magmatic reservoirs that may readily exsolve fluids and could form porphyry deposits. However, the size of these potential deposits is limited by the duration of the high magmatic flux (<~200 ka)^20^,^26^.

We focus on Andean-type porphyry Cu deposits associated with syn-subduction arc magmatism. This magmatism has a rather restricted range of Cu concentrations^18^, thus, simple mass balance considerations set a lower limit to the minimum amount of felsic-intermediate melt required to provide the Cu precipitated in a porphyry deposit (see also refs 6 and 19). This implies that the larger the amount of Cu deposited, the larger must be the volume of magma that delivered the mineralising fluids. In order to calculate volumes of intermediate to felsic melts we have used the thermal models developed in refs 16 and 20. Specifically, the petrologic model of ref. 16 is used to quantify the volumes of intermediate to felsic magmas generated through time by concomitant fractional crystallization of parent hydrous basalts and partial melting of host rocks (amphibolites at lower crustal levels and graywackes at upper crustal ones) at different crustal levels. We considered a typical long-term arc magma flux of 0.0009 km^3^/a into magmatic systems located at depths ranging from 5 to 30 km (corresponding to pressures between 0.15 and 0.9 GPa: Fig. 1). The model results presented in ref. 20 are used to quantify volumes of intermediate to felsic melts generated at shallower depths (6–10 km corresponding to 0.18–0.3 GPa) by a higher magma flux of 0.016 km^3^/a (Fig. 1). These two end-member situations encompass the conditions and rates of magma transfer in the arc crust reasonably well.

Following the model of ref. 16 we simulate injection of mantle-derived basaltic melt as circular sills of 50 m thickness and 7.5 km radius every 10 ka at depths ranging between 5 and 30 km over timescales of up to several Ma. The injected basaltic melt cools and crystallizes at a rate that is inversely proportional to the depth of emplacement (assuming a geothermal gradient of 20 °C/km in the model). Progressive magma injection leads to a temperature increase of the surrounding rocks until a residual melt (from fractionation of the injected basalt) starts to accumulate. Eventually, the temperature of the magmatic system may reach values appropriate for partial melting of the surrounding rocks and a crustal melt starts to accumulate and mix with the residual melt (from basalt fractionation), forming a hybrid melt.

The most important results of the modelling are: 1) the mass of residual melt increases with the rate and duration of magma injection; 2) injected basaltic melts cool increasingly rapidly at shallower levels in the Earth’s crust and partial melting of host rocks becomes increasingly difficult and less efficient, because the temperature of the host rocks steadily decreases with decreasing depth according to the geothermal gradient (20 °C/km). Therefore, melt productivity (the amount of hybrid melt accumulated after a certain time since the onset of the injection process with respect to the amount of total injected basaltic melt: Supplementary Information 2) decreases with decreasing depth; 3) over time, hybrid melts have compositions increasingly closer to that of the recharging basaltic magma, which is the opposite of what happens during the fractionation of a single magma batch^21^.

The model of ref. 20 is parameterised to evaluate melt productivity in shallow magmatic systems under short-lived (up to 200 ka), high magma fluxes (melt injection rate of 50 mm/a equivalent to 0.016 km^3^/a for a disk-shaped pluton with 10 km radius). The modelling approach is the same as the scenario for mantle-derived basaltic melts, with the exception that the injected magma is dacitic in composition and no partial melting of the host rocks occurs.

### H_2_O content of the hybrid melts and Cu contents of their fluids

We calculated the H_2_O concentration of hybrid melts for both scenarios assuming that H_2_O in the parent basaltic melt varies between 2 and 4 wt.% and in the host crustal rocks between 0.2 and 1 wt.% (Table 1). While we considered a fluid phase consisting solely of H_2_O, we took into account the variation of H_2_O solubility in silicate melts with pressure and chemical composition^17^ (Supplementary Information 2).

The amount of fluid needed to transport and precipitate Cu depends on the concentration of Cu in such a fluid. To calculate the concentration of Cu in the fluid phase, we first considered a range of Cu contents of magmas typical for thick volcanic arcs^17^ (Supplementary Information 2), and then we performed Monte Carlo simulations for a range of fluid-melt partition coefficients for Cu between 2 and 100 (e.g., ref. 22). We assume that there is no recycling of pre-existing sulphide cumulates, or, if there is through partial melting and incorporation into the hybrid melt, this is computed in the Cu budget of arc magmas with normal Cu concentrations^18^. The Cu concentrations in the exsolvable fluid are on the order of a few hundreds of ppm (up to 700 ppm). This compares well with the Cu concentrations measured in intermediate density fluid inclusions from porphyry systems, also taking into account the high diffusivity of Cu in quartz, which might be responsible for a post-entrapment enrichment of measured Cu in intermediate density S-bearing fluid inclusions^23^.

### Chemical composition of the hybrid melts

We have used an empirical relationship between melt productivity and SiO_2_ to determine the SiO_2_ content of the hybrid melt (see Supplementary Information 2 for further details). To calculate the Sr/Y ratios of the hybrid melts we have used available experimental petrology data^24^,^25^ on hydrous basaltic parental melts undergoing fractional crystallizion at pressures ranging from <0.1 to 1.2 GPa. The mineralogy as well as the proportions of minerals, and thus the bulk partition coefficients of Sr and Y, change both with pressure and residual melt fractions. For the fractionating minerals of the above experiments (olivine, spinel, plagioclase, orthopyroxene, clinopyroxene, amphibole, and garnet) we pooled all the available mineral/melt partition coefficients for Sr and Y (GERM database: https://earthref.org/KDD/) subdividing them according to melt composition (equivalent to residual melt fractions in a process of fractional crystallization). The median values of the partition coefficients for both elements and for every mineral display systematic changes with the composition (or residual fraction) of the melt (Supplementary Information 2 and Dataset 1). We fit the partition coefficients as a function of residual melt composition and model their contribution to the evolution of Sr/Y in the residual melt as a function of pressure and amount of melt (Supplementary Information 2 and Dataset 1).

### Monte Carlo simulations

In the Monte Carlo simulations initial H_2_O and Cu content of the melt, fluid-melt partition coefficients for Cu, duration of sill injection at depth (t), and depth of magma accumulation (P) were contemporaneously and randomly varied within specified ranges for the two selected magma fluxes (Table 1; Supplementary Information 3, Dataset 2). We performed more than 100,000 simulations.

## Results and Discussion

### Melt injection at shallow crustal levels

**Figure 2:**
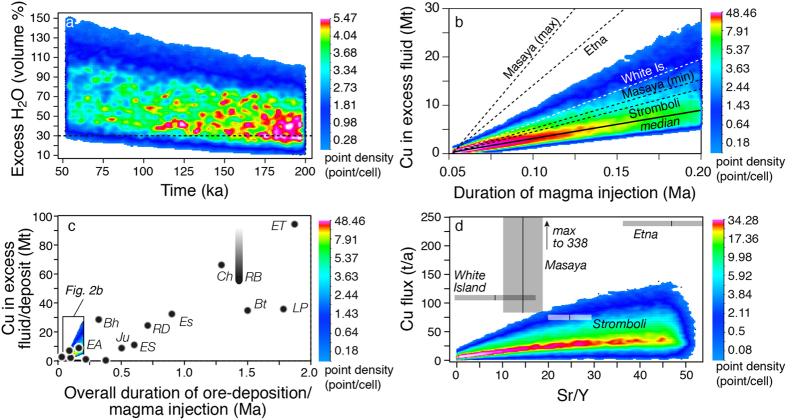
Excess H_2_O, metal budget, temporal and geochemical parameters of porphyry Cu deposits and passively degassing volcanoes. (**a**) Monte Carlo simulations of volume % of excess H_2_O in magmas accumulated under a high flux rate (0.016 km^3^/a) between 6 and 10 km depth versus duration of magma injection parameterized from ref. 20. Different colours indicate variable point densities in our simulations as shown by the scale bar on the right-hand side; (**b**) Cu (Mt) in excess fluid versus duration of magma injection for the high flux rate (0.016 km^3^/a) scenario. The continuous black line is the median value of the Cu emission rate of our simulations. The dashed black lines are projections of average Cu emission rates from passively degassing volcanoes (Etna^29^, Masaya^30^, White Island^31^, Stromboli^32^); (**c**) Cu (Mt) in porphyry copper deposits versus overall duration of the ore deposition periods (black dots) compared to the Cu (Mt) liberated by excess fluids of Fig. 2b during magma accumulation with a high magma flux (0.016 km^3^/a). The black dots representing porphyry Cu deposits are shaded to indicate that uncertainties exist on the determinations of both Cu endowments and overall duration of the ore deposition periods (see text). Cu endowment of Rio Blanco is much higher (up to >200 Mt Cu) if the whole district is taken into account (represented by shaded projection towards high Cu endowment). For abbreviations of PCD see Table S1.1; (**d**) Cu flux (tons/year) versus Sr/Y ratios of the four passively degassing volcanoes of Fig. 2b compared to our model simulations. Sr/Y values are averages from the georoc database (http://georoc.mpch-mainz.gwdg.de/georoc/) ±1σ uncertainties.

High magma fluxes (0.016 km^3^/a) at shallow crustal depths (6–10 km) may result in the assembly of large, transient (≤~200 ka), intermediate-felsic magmatic reservoirs^20^,^26^. Our results show that, under these conditions, magmas are H_2_O-saturated throughout the period of magma accumulation with more than 30 vol.% of excess fluid in >90% of the simulations, which is sufficient for a continuous extraction of fluids^27^, potentially Cu-bearing (Fig. 2a). Assuming that these extracted fluids generate mineralization^28^, the median value of Cu accumulation after the maximum lifetime (~200 ka) permissible for such a sustained flux^20^,^26^ would be <9 Mt (<4.5 Mt Cu for 50% efficiency: Fig. 2b). This could be a viable mechanism to form at best deposits with <~5 Mt Cu (50% efficiency), but not larger ones (Fig. 2c). Interestingly, the Cu exsolution rates and Sr/Y values calculated for these short-lived magmatic systems are comparable to Cu emission rates and Sr/Y compositions of persistently degassing volcanoes (Etna^29^, Masaya^30^, White Island^31^, Stromboli^32^; Fig. 2d).

**Figure 3:**
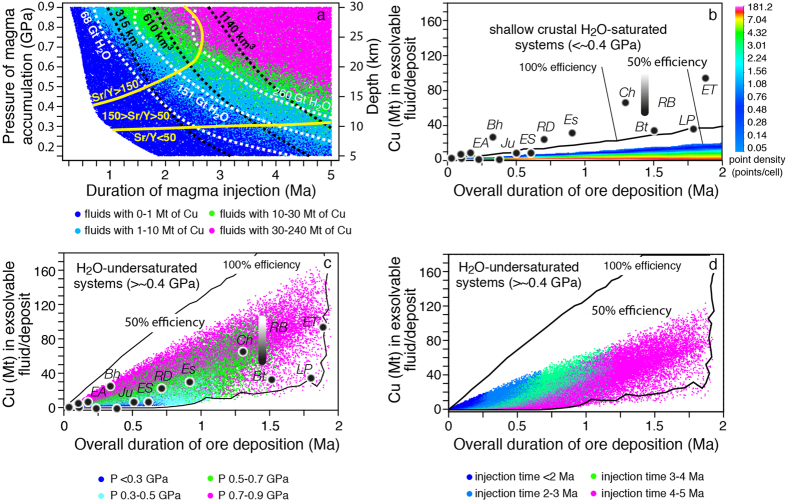
Monte Carlo simulations of porphyry Cu productive and non-productive magmatic systems under an average arc magma flux of 0.0009 km^3^/a. (**a**) Pressure versus time conditions for the generation of magmatic systems with different Cu potential endowments (white and black dashed lines indicate the Gt of H_2_O dissolved in the hybrid melt and hybrid melt volume in km^3^, respectively; yellow lines represent limits of Sr/Y values of hybrid melts resulting from our simulations); (**b**) Monte Carlo simulations of Cu (Mt) in excess fluid versus duration of magmatic cycle for H_2_O-saturated magmatic systems formed at crustal depths <~0.4 GPa. The black dots represent radiometrically measured timescales of porphyry Cu deposits with respect to their endowment (Table S1.1). The onset of the mineralization has been placed in correspondence with the beginning of magma accumulation to allow comparison of the two timescales. For abbreviations of PCD see Table S1.1; (**c**) Cu (Mt) in the exsolvable fluid of hybrid melts after injection times of 0 to 5 Ma versus the overall duration of the ore deposition period. Color classes indicate different pressures of magma accumulation; (**d**) same as (**c**) but with colour codes indicating different times of magma accumulation.

Mafic magma injection under average arc magma flux (0.0009 km^3^/a) at shallow crustal levels (P < ~0.4 GPa) prevents the accumulation of significant amounts of hybrid melt and dissolved Cu-bearing fluids (Cu < 1 Mt even after 3 Ma: Fig. 3a) because of the large amount of heat lost to the relatively cool host rock at shallow crustal levels^16^. The rate of potential Cu-release by these magmatic systems is significantly lower than the rates at which Cu is precipitated in porphyry systems (all Cu endowments of porphyry deposits are much higher for a given time than our model results in Fig. 3b). This indicates that shallow level accumulation and fractionation of mafic magmas under average arc fluxes is not a viable mechanism to generate giant porphyry deposits in the timeframes measured in natural deposits.

### Basaltic melt injection at mid- to deep crustal levels

Basaltic melt injection at pressures >0.4 GPa (>~13 km) and with an average arc magma flux (0.0009 km^3^/a) results in a higher hybrid melt productivity because of the higher temperature of the surrounding rocks^16^ (see km^3^ of hybrid melt in Fig. 3a). Additionally, the H_2_O content of the hybrid melt is higher (Fig. 3a) because of the strong dependence of fluid solubility on pressure^17^. Therefore, basaltic melt injection at pressures >0.4 GPa over times >3 Ma, leads to the accumulation of hybrid melt containing sufficient H_2_O to deliver up to 10 Mt Cu (Fig. 3a). At accumulation pressures >0.5 GPa and accumulation times >3.5 Ma, the hybrid melt contains enough H_2_O to deliver >30 and up to 240 Mt Cu (Fig. 3a).

Our simulations indicate that magmatic systems capable of forming porphyry deposits with >10 Mt Cu must develop at mid- to lower crustal depths (>~17 km, P > 0.5 GPa) and require accumulation times >2.5 Ma with an average arc magma flux (Fig. 3a,c,d). The modelling results are in agreement with the evidence that large porphyry systems develop at the end of magmatic cycles lasting several Ma^33^,^34^,^35^,^36^ (2–5 Ma; Supplementary Information 1) with average magma fluxes^37^.

### Transfer of magma from the deep accumulation reservoir to shallower crustal levels

**Figure 4:**
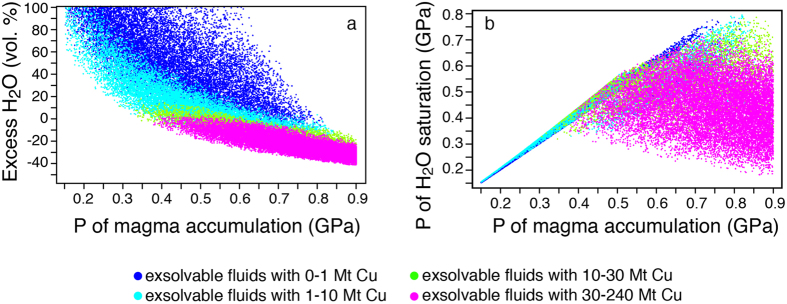
Monte Carlo simulations of the volume % of excess H_2_O and pressure of H_2_O saturation versus pressure of accumulation of the magmatic system. (**a**) Simulations of volume % of excess H_2_O versus pressure of accumulation. Negative values indicate undersaturation and its degree (percentages are calculated with respect to melt volume). The colour codes correspond to different amounts of Cu potentially delivered by magmatic systems. The potentially most productive systems (bright pink dots) accumulate at P > 0.4 GPa and at these pressures of accumulation they are always H_2_O-undersaturated; (**b**) simulations of pressures of accumulation of magmatic systems versus pressures at which the same systems become H_2_O-saturated. The coloured dots correspond to magmatic systems able to deliver different amounts of Cu. Whereas the less productive magmatic systems plot along a ~45° slope meaning that they are H_2_O-saturated or nearly so at the depth of their accumulation, the most productive systems plot along a subhorizontal trend meaning that they are H_2_O-undersaturated. The majority of these systems reach H_2_O saturation at P between 0.25 and 0.55 GPa (i.e., depths of 8 to 18 km).

Our results suggest that magmas containing the largest amounts of H_2_O and Cu (>30 Mt) are systematically water-undersaturated because they formed at high pressures (>0.5 GPa; Fig. 4a). Therefore, fluid- and Cu-bearing magmas accumulated in the lower to middle crust may rise towards shallower levels carrying the bulk of their water and Cu, before starting to release them upon reaching fluid saturation pressures. Calculations show that if these magmas rose adiabatically, the majority of the simulated fertile magmas would become H_2_O-saturated at pressures between 0.25 and 0.55 GPa (Fig. 4b). This pressure interval corresponds to depths of ~8–18 km, which are in the same range of the main magmatic reservoirs inferred for several porphyry-type deposits from geophysical, geochemical and mineralogical data^1^,^6^,^19^,^38^.

Thus, the formation of large accumulations of hybrid melts at mid- to lower crustal levels is an essential step in the formation of the porphyry system which sets the upper size limit of the potential deposit. However, it is the next step (the transfer of this large accumulation of hybrid melt to shallower levels from where fluids can be exsolved) that determines the formation and the ultimate size of the deposit (see also ref. 28). This is suggested by data on Cu endowment and overall duration of the ore deposition period for different deposits (Supplementary Information 1, Table S1.1). Here, the overall duration of the ore deposition period was bracketed using only Re-Os dating of molybdenite from different hydrothermal pulses and/or U-Pb zircon dating of syn- and late/post-mineral porphyries (Table S1.1). Although the measurements of both Cu endowment and ore deposition period are subject to large uncertainties related to methodological approaches, uncertainties in cross-calibration of dating techniques and sampling bias, a broadly positive correlation exists between Cu endowment and the overall duration of the ore deposition period (Fig. 2c). Therefore, it is reasonable to consider that Cu in most porphyry deposits is precipitated at a long-term average rate of a few tens (~40) of tons of Cu per year. This does not mean that Cu is continuously precipitated at this rate. Rather, as pointed out by several geochronological studies^9^,^10^,^39^, this suggests that larger deposits are formed by a higher number of short-lived (few tens of ka) pulses distributed over a longer timescale than in smaller deposits, with the long-term average rate of Cu deposition being similar in both cases. The overall longer lifetime of magmatic-hydrothermal activity in the larger deposits could be due either to a larger availability of hybrid melt in the deep reservoir of the larger deposits or to earlier tectonic interruption of the hybrid melt transfer from depth for the smaller deposits. The latter seem to be characterized by durations of precursor magmatic activity as long as those in the largest deposits (Supplementary Information 1). Hence, it is possible that tectonics play an important role in modulating the transfer of magma from the deep to the shallow reservoir.

### Sr/Y composition of magmatic rocks associated with porphyry Cu deposits

**Figure 5:**
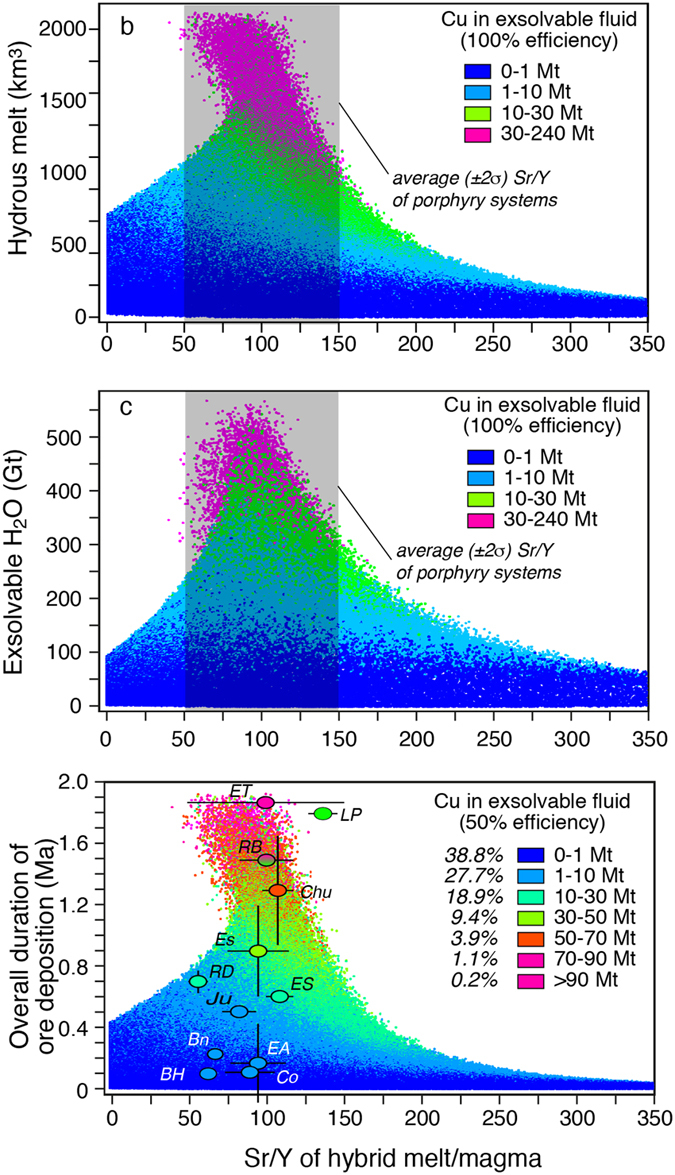
Monte Carlo simulations of the overall ore deposition periods, volumes, H_2_O contents and Sr/Y values of the hybrid melt. (**a**) Volume of accumulated hybrid melt at various crustal levels versus its Sr/Y composition. The largest systems (and most fertile in terms of exsolvable Cu) correspond to Sr/Y values that overlap with the average (±2σ) Sr/Y values (grey box) of major porphyry Cu deposits (Table S1.1; (**b**) giga-tons of exsolvable H_2_O contained in the hybrid melts accumulated at different crustal depth versus their Sr/Y compositions. Again, the systems containing more H_2_O (and most fertile in terms of exsolvable Cu) correspond to Sr/Y values that overlap with the average (±2σ) Sr/Y values of porphyry Cu deposits (Table S1.1). The colour codes correspond to different amounts of Cu potentially delivered by the magmatic systems; (**c**) Simulations of overall durations of the ore deposition periods versus Sr/Y values of associated magmas subdivided by classes of exsolvable Cu (Mt) with 50% efficiency (half of total exsolvable Cu). The model data (except Los Pelambres) show a good fit with natural porphyry system data (coloured circles: the colors of the circles indicate which Cu tonnage class reported in the legend each deposit belongs to; Rio Blanco is bicolor because, taking into account the whole district, Cu endowment may rise to >200 Mt Cu).

**Figure 6:**
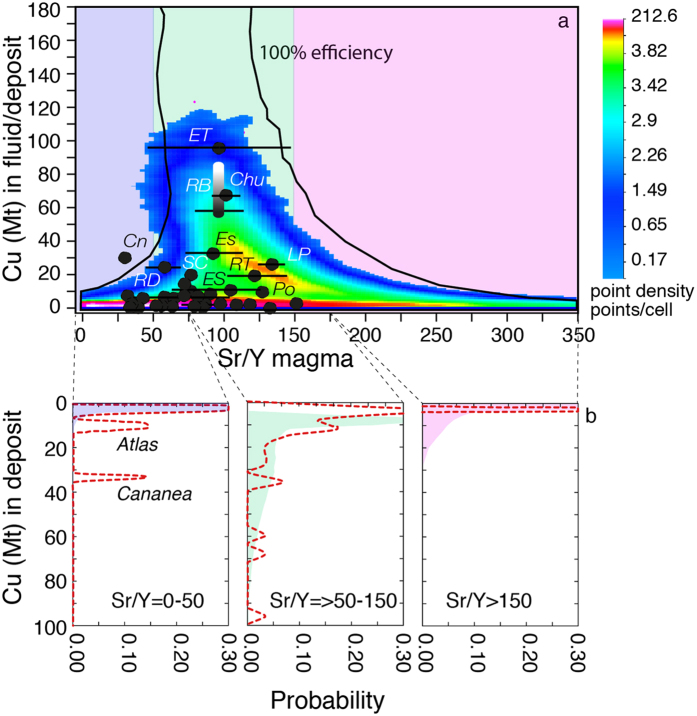
Monte Carlo simulations of magmatic system productivity with respect to their Sr/Y values. (**a**) Cu Mt in fluid exsolvable (50% efficiency) from magmatic systems formed at various depths (5–30 km) and after different injection times (0–5 Ma) versus hybrid melt Sr/Y. Also reported are the Cu endowments and average Sr/Y values (±1σ) of porphyry Cu deposits for which data are available (Table S1.1). For abbreviations of PCD see Table S1.1; (**b**) Probability distribution of magmatic system productivity for the three intervals of Sr/Y values determined by the average ±2σ (100 ± 50) values of natural porphyry Cu deposits (i.e., <50, >50 and <150, >150). The red dashed lines show the probability distributions for natural porphyry deposits (Table S1.1). The peaks at about 10 and 30 Mt of Cu for Sr/Y < 50 are due to the Atlas and Cananea deposits, for which limited Sr/Y values are available (1 and 2 respectively: Table S1.1).

Our modelling results quantitatively account for the association between supergiant porphyry Cu deposits (>10 Mt of Cu at 50% efficiency) and magmas with Sr/Y values between ~50 and ~150^12^ (Figs 5 and 6). In fact, in our model this range of natural Sr/Y values associated with major porphyry copper deposits corresponds to timescales (t) and crustal levels (P) of magma accumulation that are the most favourable for the development of the largest and H_2_O-richest magma volumes (Figs 3a and 5a,b). The availability of such a large amount of H_2_O-rich magma (with its corresponding “diagnostic” Sr/Y values) allows a long-lived transfer to shallower levels from where Cu can be released to form mineralization.

Our model suggests, in agreement with natural porphyry data, that for both higher (>150) and lower (<50) Sr/Y values, the probability to form deposits with >10 Mt Cu is extremely low (Fig. 6), because total amounts of magmas and associated H_2_O formed at the corresponding P-t conditions are not sufficiently large (Figs 3a and 5a,b). Such limited H_2_O and magma quantities are not sufficient to sustain the longer-lived ore deposition period required to form supergiant deposits (Fig. 5c). In contrast, for magmatic systems with Sr/Y between 50 and 150 the probabilities to deliver 30–50 and 70–90 Mt Cu (50% efficiency) are ~9 and 4% of the simulations, respectively (Figs 5c and 6). The probability to form a deposit with >90 Mt Cu (El Teniente-type) drops to about 0.2% (Figs 5c and 6). Additionally, the largest number of simulations (66.5%) results in the production of porphyry deposits with <10 Mt Cu, which is also observed in natural deposits (Fig. 6). The remarkable fit between model predictions and natural porphyry data (Figs 5c and 6) suggests that our model (based on measurable parameters like the overall duration of ore deposition period and average Sr/Y values of associated magmatic rocks; Fig. 5c) could be used for preliminary evaluation of mineral resources during early stages of mineral exploration.

## Conclusions

Our results suggest that the formation of arc magmatic systems associated with porphyry Cu deposits occurs in two steps: 1) long-lasting (>~2.5 Ma) injection of hydrous basalts in the mid- to lower crust (>~17 km) leading to the formation of large amounts (>800 km^3^) of andesitic magma (SiO_2_ = 57–64 wt.%) with a specific interval of Sr/Y ratios (50–150) and high H_2_O concentrations (85% of the simulations of the most fertile magmas, with exsolvable Cu > 30 Mt, are between 5.5–13 wt.% H_2_O; Supplementary Information 4, Figure S4.1) under an average magmatic arc flux (e.g., 0.0009 km^3^/a); 2) subsequent transfer of this magma to mid-/upper crustal levels (~8–18 km), from where magma may provide copper-bearing fluids, during recurring episodes of mineralization lasting up to ~2 Ma. Whereas the first step (formation of large accumulation of hybrid melts at mid- to lower crustal levels) sets the upper size limit of the potential deposit, it is the next step (duration of the transfer of this large accumulation of hybrid melt to shallower levels from where fluids can be exsolved) that seems to determine the ultimate size of the deposit. The occurrence of these two steps also provides an explanation for the observed transition of tectonic regimes associated with the formation of giant porphyry systems in magmatic arcs, sometimes accompanied by a magmatic lull, from a mainly compressional stage (favouring multi-Ma accumulation of magma at depth) to a near neutral stress or slightly extensional stage^38^,^40^ (promoting the migration of magma towards shallower depths and fluid exsolution within the timescales of porphyry deposit formation). The termination of the magma transfer during this second step could be due either to exhaustion of the mid-crustal magma reservoir or, more likely, to external tectonic processes. Magmatic systems that do not grow enough in size at depth cannot sustain the long-lived magmatic-hydrothermal activity that is recorded by the largest porphyry systems.

Overall, while not discounting the potential role of specialized magmas^18^,^41^, our model suggests that typical arc processes and melt compositions are able to explain the wide range of Cu endowments observed in porphyry deposits.

## Methods

Our model uses a Monte Carlo approach to simulate (>100000 simulations) the amounts of hybrid melt produced in the crust (melt productivity = amount of hybrid melt accumulated/amount of total intruded basaltic melt), their water contents (both in solution and exsolvable), the Cu contents in the exsolvable water and the SiO_2_ as well as Sr/Y compositions of the hybrid melts produced within the crust.

### Melt productivity

We quantify melt productivity at different crustal depths under (i) an average arc magma flux [5 mm/a of basaltic melt injection rate through a circular section of 7500 m of radius, equivalent to 0.0009 km^3^/a] and (ii) an episodically high magma flux [50 mm/a injection rate of dacitic melt through a circular section with 10000 m radius, equivalent to 0.016 km^3^/a], using the models of refs 16 and 20.

For the average arc magma flux (0.0009 km^3^/a) we parameterized melt productivity for a time interval between 0 and 5 Ma and for pressures between 0.15 and 0.9 GPa (corresponding to crustal depths of ~5 to ~30 km)^16^.

We allowed pressure (P) and time (t) of maturation of the magmatic systems to vary randomly within the above mentioned fixed limits (Table 1) to obtain, for any random value of P and t, the corresponding value of melt productivity using a Monte Carlo method.

We have parameterized the curves of melt productivity from ref. 16 for both residual (M_residual_) and crustal melt (M_crustal_) fractions (Supplementary Information 2, Figure S2.1), which are expressed as polynomial functions of pressure (P) of the type


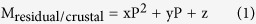


where M is the residual or crustal melt fraction, P is the pressure at which injection and accumulation of residual/crustal melt is occurring and x, y and z are variables that depend on the incubation time through best fit polynomial equations of the type


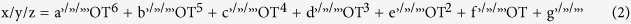


where OT is the time since the onset of the injection of basaltic magma and a^’/”/”’^, b^’/”/”’^, c^’/”/”’^, d^’/”/”’^, e^’/”/”’^, f^’/”/”’^, g^’/”/”’^ are constant values different for each one of the x, y and z variables.

For the high magma flux (0.016 km^3^/a) scenario we parameterized melt productivity for a time interval up to 0.2 Ma (a maximum estimate for a continuously high magma flux^20^) and for depths of magma emplacement shifting steadily through time from 6 to 10 km due to continuous injection of the sills underneath the previous ones^20^ (Supplementary Information 2, Figure S2.2). Volume of melt is calculated from volume of mobile magma assuming that the melt fraction in the mobile magma ranges randomly between 60 and 80% of the magma volume^20^.

### H_2_O concentrations in the hybrid melt

Melt productivity as determined above was coupled with H_2_O concentrations in the hybrid melt assuming a geologically sound random range of initial H_2_O contents in the mantle-derived basalt (2–4 wt.%) and in the amphibolitic (lower) to greywacke (upper) crust (0.5–1.0 wt.%) and assuming a completely incompatible behaviour of H_2_O during the hybrid melt accumulation process. We then used VolatileCalc^17^ to calculate water solubility in melts according to pressure of accumulation and hybrid melt composition (Supplementary Information 2, Figure S2.3). This way we could determine the H_2_O contents of the melts and the degree of H_2_O over- or under-saturation in the hybrid melts produced at different crustal levels (P) and after different durations of injection. This allowed us to determine how much H_2_O was associated with any specific hybrid melt produced after any injection time, at any crustal depth and under different magma fluxes. At any specified pressure, H_2_O solubility is linked to melt fraction (M) by a best-fit polynomial equation (Supplementary Information 2, Figure S2.4) of the type


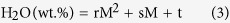


where M = melt fraction and r, s, t are pressure-dependent variables according to other 2^nd^ order polynomial equations (Supplementary Information 2, Figure S2.5) of the type


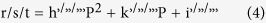


where h^’/”/”’^, k^’/”/”’^, i^’/”/”’^ are constant values specific to each one of the r, s, t variables. Combining these equations allows us to reproduce for any random P and M the solubility of H_2_O to be used in the Monte Carlo simulations.

### Cu concentrations in the exsolved fluid

The amount of fluid (and consequently of melt) needed to transport and precipitate Cu as observed in natural deposits is dependent on the concentration of Cu in such a fluid. The latter depends on the Cu concentration in the melt and on the value of the fluid-melt partition coefficient, which determines how much Cu goes into the fluid once it separates from the melt. For Cu concentrations in the hybrid melt we use the composition-(SiO_2_−) dependent Cu concentrations of continental arc magmas of ref. 18. The SiO_2_-Cu relationship is best fitted by a second order equation (y = ax^2^ + bx + c, where y = Cu (ppm) and x = SiO_2_) that expresses the covariation between median Cu and SiO_2_ values from thick arc magmas (>30 km) (ref. 18) (Supplementary Information 2, Figure S2.6). Since there is some scatter in the SiO_2_-Cu relationship we consider all possible values within the upper and lower boundaries of this scatter (also defined by second order polynomial equations in Figure S2.6) and implement them in the Monte Carlo modelling. For Cu KDs we use a conservative approach according to which we attribute a random variation of Cu fluid-melt KDs between 2 and 100 for any type of hybrid melt produced.

### Hybrid melt composition (SiO_2_ and Sr/Y)

In order to link the melt productivity of the model of ref. 16 to a chemical composition (SiO_2_) we use the relationship below (Supplementary Information 2, Table S2.1) between melt fraction and SiO_2_ based on the mid-values of SiO_2_ for the fields of basalt, basaltic andesite, andesite, dacite and rhyolite of the TAS diagram^42^ and the mid-values of the melt fraction and the corresponding composition attributed by ref. 16 (e.g., Figure 8 of ref. 16).

In a bivariate plot, the two variables above are linked through the equation


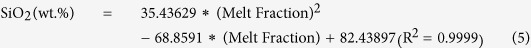


Finally we calculate Sr/Y values of melt fractions based on: (i) statistical treatment of available mineral-melt KD values of Sr and Y (GERM database: https://earthref.org/KDD/) for various minerals and their correlation with changing melt composition; (ii) experimentally-determined proportions of mineral phases during crystallization of hydrous basalts at various pressures; (iii) implementation of the changing Sr and Y KDs with melt composition and changing mineral phases with pressure to obtain Sr/Y values of hybrid melts as a continuous function of pressure and amounts of hybrid melt (see Supplementary Information 2 and Supplementary Dataset 1 for more details).

### Simulation of the overall duration of the ore deposition period

The overall duration of the ore deposition period in our simulations was obtained considering the average Cu flux rate in natural porphyry Cu deposits which is provided by the broad linear correlation (Supplementary Information 2, Figure S2.7) between Cu endowment and overall duration of the ore deposition periods for selected porphyry Cu deposits (i.e., those with the best constrained radiometric dating available: see Table S1.1 in Supplementary Information 1). Although measurements of both these parameters may be subject to large uncertainties related to methodological approaches, uncertainties in cross-calibration of dating techniques and sampling bias, the positive correlation between them (Overall duration of ore deposition = 0.0202 * Cu (Mt), R^2^ = 0.74, p < 0.00001) provides a reasonable first order approximation of the average rate at which Cu is precipitated in porphyry Cu deposits of variable sizes (~40 tons of Cu per year). This rate is used to quantify the time that is needed to flux the Cu contained in the exsolvable fluid associated with the variable amounts of hybrid melts produced by our simulations, using the equation


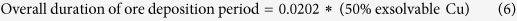


assuming that 50% of the exsolved Cu is actually deposited.

### Molar volume of H_2_O

The molar volume of H_2_O at different pressures (0.2, 0.3, 0.4, 0.5, 0.6, 0.7, 0.8 GPa) and at a temperature of 950 °C (typical for andesitic melts) was calculated using the online fugacity calculator at “ https://www.esci.umn.edu/people/researchers/withe012/fugacity.htm” and parameterizing the variation of the molar volume of H_2_O with pressure by the following best fit equation


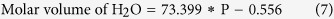


where P = pressure (in GPa). Using temperatures of ±100 °C has a minimal effect on the molar volume of H_2_O.

## Additional Information

**How to cite this article:** Chiaradia, M. and Caricchi, L. Stochastic modelling of deep magmatic controls on porphyry copper deposit endowment. *Sci. Rep.* **7**, 44523; doi: 10.1038/srep44523 (2017).

**Publisher's note:** Springer Nature remains neutral with regard to jurisdictional claims in published maps and institutional affiliations.

## Supplementary Material

Supplementary Information 1

Supplementary Information 2

Supplementary Information 3

Supplementary Information 4

Supplementary Dataset 1

Supplementary Dataset 2

## Figures and Tables

**Table 1:** Main parameters used for Monte Carlo simulations under two different magma fluxes.

Injection rate of 5 mm/a through a disk of 7500 m radius^a^ (equivalent to a magma flux of 0.0009 km^3^/a)	Injection rate of 50 mm/a through a disk of 10000 m radius^b^ (equivalent to a magma flux of 0.016 km^3^/a)
Independent (random) parameters	
• Time = 0 to 5 Ma	• Time = 51526 to 200000 years
• Pressure = 0.15 to 0.9 GPa	• Depth = 6–10 km systematically shifting through time^c^
• H_2_O in parent magma = 2 to 4 wt.%	• H_2_O in parent magma = 2 to 4 wt.%
• H_2_O in assimilant = 0.2 to 1 wt.%	• % melt in mobile magma = 61 to 80%^a^
• KD Cu fluid-melt = 2–100	• KD Cu fluid-melt = 2–100

For full set of parameters see Tables S3.1 and S3.2 in Supplementary Information 3.

^a^An average size for crustal magma chambers, typically ranging between 5000 and 10000 m (ref. 16).

^b^As modeled by ref. 20.

^c^Following ref. 20.

## References

[b1] Sillitoe R. H. Porphyry copper systems. Econ. Geol. 105, 3–41 (2010).

[b2] Richards J. P. Postsubduction porphyry Cu-Au and epithermal Au deposits: Products of remelting of subduction-modified lithosphere. Geology 37, 247–250 (2009).

[b3] Cline J. S. & Bodnar R. J. Can economic porphyry copper mineralization be generated by a “typical” calc-alkaline melt? Journal of Geophysical Research 96, 8113–8126 (1991).

[b4] Hedenquist J. W. & Lowenstern J. B. The role of magmas in the formation of hydrothermal ore deposits. Nature 370, 519–527 (1994).

[b5] Candela P. A. & Piccoli P. M. Magmatic processes in the development of porphyry-type ore systems. Economic Geology 100th Anniversary volume 25–37 (2005).

[b6] Cloos M. Bubbling Magma Chambers, Cupolas, and Porphyry Copper Deposits. International Geology Review 43/4, 285–311 (2001).

[b7] Von Quadt A. . Zircon crystallization and the lifetimes of ore-forming magmatic-hydrothermal systems. Geology 39, 731−734 (2011).

[b8] Chiaradia M., Schaltegger U., Spikings R. A., Wotzlaw J. F. & Ovtcharova M. How accurately can we date the duration of magmatic-hydrothermal events in porphyry systems? Econ. Geol. 108, 565−584 (2013).

[b9] Campbell I. H., Ballard J. R., Palin J. M., Allen C. & Faunes A. U-Pb zircon geochronology of granitic rocks from the Cuquicamata-El Abra porphyry copper belt of northern Chile: Excimer laser ablation ICP- MS analysis. Econ. Geol. 101, 1327−1344 (2006).

[b10] Deckart K. . Magmatic and hydrothermal chronology of the giant Río Blanco porphyry copper deposit, central Chile: Implications of an integrated U-Pb and ^40^Ar/^39^Ar database. Econ. Geol. 100, 905−934 (2005).

[b11] Buret Y. . From a long-lived upper-crustal magma chamber to rapid porphyry copper emplacement: Reading the geochemistry of zircon crystals at Bajo de la Alumbrera (NW Argentina). Earth Planet. Sci. Lett. 450, 120–131 (2016).

[b12] Tapster S. . Rapid thermal rejuvenation of high-crystallinity magma linked to porphyry copper deposit formation; evidence from the Koloula Porphyry Prospect, Solomon Islands. Earth Planet. Sci. Lett. 442, 206–217 (2016).

[b13] Loucks R. R. Distinctive composition of copper-ore-forming arc magmas. Austral. J. Earth Sci. 61, 5–16 (2014).

[b14] Richards J. P. High Sr/Y arc magmas and porphyry Cu ± Mo ± Au deposits: just add water. Econ. Geol. 106/7, 1075–1081 (2011).

[b15] Chiaradia M., Ulianov A., Kouzmanov K. & Beate B. Why large porphyry Cu deposits like high Sr/Y magmas? Sci. Rep. 2, n. 685 (2012). 10.1038/srep00685 PMC3449286 23008750

[b16] Annen C., Blundy J. D. & Sparks R. S. J. The genesis of intermediate and silicic magmas in deep crustal hot zones. J. Pet. 47, 505–539 (2006).

[b17] Newman S. & Lowenstern J. B. VolatileCalc: a silicate melt-H_2_O-CO_2_ solution model written in Visual Basic for Excel. Computers and Geosciences 28, 597–604 (2002).

[b18] Chiaradia M. Copper enrichment in arc magmas controlled by over- riding plate thickness. Nature Geoscience 7, 43−46 (2014).

[b19] Clark A. H. Are outsize porphyry copper deposits either anatomically or environmentally distinctive? SEG Spec. Pub. 2, 213–284 (1995).

[b20] Annen C. From plutons to magma chambers: Thermal constraints on the accumulation of eruptible silicic magma in the upper crust. Earth Planet Sci. Lett. 284, 409–416 (2009).

[b21] Melekhova E., Annen C. & Blundy J. Compositional gaps in igneous rock suites controlled by magma system heat and water content. Nature Geoscience 6(5), 385–390 (2013).

[b22] Simon A. C. . Copper partitioning in a melt–vapor–brine–magnetite–pyrrhotite assemblage. Geochim. Cosmochim. Acta 70, 5583–5600 (2006).

[b23] Lerchbaumer L. & Audétat A. High Cu concentrations in vapor-type fluid inclusions: An artifact? Geochim. Cosmich. Acta 88, 255–274 (2012).

[b24] Moore G. & Carmichael L. S. E. The hydrous phase equilibria (to 3 kbar) of an andesite and basaltic andesite from western Mexico: constraints on water content and conditions of phenocryst growth. Contrib. Mineral. Petrol. 130, 304–319 (1998).

[b25] Müntener O., Kelemen P. O. & Grove T. L. The role of H_2_O during crystallization of primitive arc magmas under uppermost mantle conditions and genesis of igneous pyroxenites: an experimental study. Contrib. Mineral. Petrol. 141, 643–658 (2001).

[b26] Schöpa A. & Annen C. The effects of magma flux variations on the formation and lifetime of large silicic magma chambers. Journal of Geophysical Research: Solid Earth 118, 926–942 (2013).

[b27] Rintoul M. D. & Torquato S. Precise determination of the critical threshold and exponents in a three-dimensional percolation model. J. Phys. A: Math. Gen. 30, L585–L592 (1997).

[b28] Chelle-Michou C., Rottier B., Caricchi L. & Simpson G. Tempo of magma degassing and the genesis of porphyry copper deposits. Sci. Rep. 7, n. 40566 (2017). 10.1038/srep40566 PMC5227963 28079160

[b29] Calabrese S. . Atmospheric sources and sinks of volcanogenic elements in a basaltic volcano (Etna, Italy). Geochim. Cosmochim. Acta 75, 7401–7425 (2011).

[b30] Moune S., Gauthier P.-J. & Delmelle P. Trace elements in the particulate phase of the plume of Masaya Volcano, Nicaragua. J. Volcanol. Geotherm. Res. 193, 232–244 (2010).

[b31] Wardell L. J., Kyle P. R. & Counce D. Volcanic emissions of metals and halogens from White Island (New Zealand) and Erebus volcano (Antarctica) determined with chemical traps. J. Volc. Geotherm. Res. 177, 734–742 (2008).

[b32] Allard P. . Acid Gas and Metal Emission Rates during Long-lived Basalt Degassing at Stromboli Volcano. Geophysical Research Letters 27/8, 1207–1210 (2000).

[b33] Rohrlach B. D. & Loucks R. R. Multi-million-year cyclic ramp-up of volatiles in a lower crustal magma reservoir trapped below the Tampakan Cu–Au deposit by Mio-Pliocene crustal compression in the southern Philippines. In Porter T. M. (Ed) Super Porphyry Copper & Gold Deposits - A Global Perspective PGC Publishing, Adelaide, v. 2, pp. 369–407 (2005).

[b34] Chiaradia M., Merino D. & Spikings R. Rapid transition to long-lived deep crustal magmatic maturation and the formation of giant porphyry-related mineralization (Yanacocha, Peru). Earth Planet. Sci. Letters 288, 505−515 (2009).

[b35] Chelle-Michou C., Chiaradia M., Ovtcharova M., Ulianov A. & Wotzlaw J.-F. Zircon petrochronology reveals the temporal link between porphyry systems and the magmatic evolution of their hidden plutonic roots (the Eocene Coroccohuayco deposit, Peru). Lithos 198–199, 129–140 (2014).

[b36] Stern C. R., Skewes M. A. & Arévalo A. Magmatic Evolution of the Giant El Teniente Cu-Mo Deposit, Central Chile. J. Pet. 52, 1591–1617 (2010).

[b37] Caricchi L., Simpson G. & Schaltegger U. Zircons reveal magma fluxes in the Earth’s crust. Nature 511, 457–461 (2014). 25056063 10.1038/nature13532

[b38] Richards J. P. Tectono-magmatic precursors for porphyry Cu-(Mo-Au) deposit formation. Econ. Geol. 98, 1515−1533 (2003).

[b39] Mercer C. N., Reed M. H. & Mercer C. M. Time scales of porphyry Cu deposit formation: insights from titanium diffusion in quartz. Econ. Geol. 110, 587–602 (2015).

[b40] Bertrand G., Guillou-Frottier L. & Loiselet C. Distribution of porphyry copper deposits along the western Tethyan and Andean subduction zones: Insights from a paleotectonic approach. Ore Geology Reviews 60, 174–190 (2014).

[b41] Lee C.-T. A. . Copper systematics in arc magmas and implications for crust–mantle differentiation. Science 336, 64–68 (2012). 22491850 10.1126/science.1217313

[b42] Le Bas M. J., Le Maitre R. W., Streckeisen A. & Zanettin B. A chemical classification of volcanic rocks based on the total alkali-silica diagram. J. Pet. 27, 745–750 (1986).

