# Supplementary material for: Stochastic modelling of deep magmatic controls on porphyry copper deposit endowment
Source: Sci Rep. 2017 Mar 15;7:44523. doi: 10.1038/srep44523 (PMC5353633; doi:10.1038/srep44523)
Supplement: Supplementary Information 1 [file srep44523-s1.pdf]

Supplementary Information 1

Table S1.1

Stochastic modelling of deep magmatic controls on porphyry copper deposit endowment

Massimo Chiaradia*, Luca Caricchi

Department of Earth Sciences, University of Geneva, Rue des Maraîchers 13, 1205 Geneva, Switzerland

**Corresponding author: Tel.: +41 22 379 66 34; Fax: +41 22 379 32 10; e-mail: Massimo.Chiaradia@unige.ch*

Table S1.1: Data relative to porphyry copper deposits available from the literature (average Sr/Y of the magmatic rocks associated with the deposits, duration of the mineralising event, duration of the magmatic cycle). Uncertainties (1σ) for Sr/Y are the standard deviations for the population of Sr/Y values available. Durations of the mineralising event and magmatic cycle are the differences between the oldest and youngest ages pertinent to these geological processes with their propagated uncertainties where uncertainties on the above ages are available.

**Deposit**	**Acronym**	**Province**	**Sr/Y average**	**1σ**	**N. of Sr/Y values**	**Cu (Mt)**	**Overall duration of ore period (Ma)**	**1σ (Ma)**	**Magma cycle minimum duration (Ma)**	**1σ (Ma)**	**Ref. Sr/Y**	**Ref. Cu Mt**	**Ref. duration of ore period**	**ref. magma cycle duration**
Craigmont	Cg	Canada (BC)	41.7	1.0	2	0.6					1	5		
El Abra	EA	Chile (Central)	95.0	17.8	5	8.8	0.16	0.26	4.65	0.38	1	6	21	21
El Teniente	ET	Chile (Central)	99.9	50.6	11	94.4	1.88	0.036	4.48		1	7	22,23	35
Rio Blanco	RB	Chile (Central)	100.4	17.3	2	56.7	1.44	0.036	3.48		1	7	24	24
Los Pelambres	LP	Chile (Central)	136.8	9.4	15	36.0	1.79	0.059	3.68	0.1	1	7	25	36,37
Potrerillos	Po	Chile (North.)	130.5	-	1	10.1					1	6		
Escondida	Es	Chile (North.)	95.1	19.8	3	32.5	0.9	0.3	4.2	1	1	7	26	38,37
Chuquicamata	Chu	Chile (North.)	107.7	10.1	6	66.4	1.3	0.36	3.5	0.4	1	7	27	39,37
El Salvador	ES	Chile (North.)	108.9	8.7	6	11.3	0.6		3		1	7	28	40
Radomiro Tomic	RT	Chile (North.)	122.3	20.4	8	19.9					1	7		
Chaucha	Cha	Ecuador				0.6	0.38	0.064	5.21		2	8	29	2,41
Junin	Ju	Ecuador	82.8	10.9	3	8.7	0.5	0.036	4.000		2	9	29	2,41
Batu Hijau	BH	Indonesia	62.8	-	1	7.2	0.09	-			1	7	30	
Sungun	Su	Iran	41.6	-	1	3.8					1	10		
Sar Cheshmeh	SC	Iran	76.9	28.0	15	14.4					3	7		
Cananea	Cn	Mexico	34.5	-	1	30.0					1	7		
Kharmagtal	Kh	Mongolia	59.4	-	1	0.8					1	6		
Cro Colorado	CC	Panama	79.2	-	1	20.3					1	7		
Yandera	Ya	P. N. Guinea	120.8	2.6	2	2.4					1	11		
Coroccohuayco	Co	Peru (South.)	89.7	16.0	19	3.0	0.1	-	2.4		4	12	31	42
Antapaccay	Ay	Peru (South.)	111.6	-	1	3.2			5.2	0.7	1	12		43,37
Cro Verde	CV	Peru (South.)	77.2	0.7	2	15.7					1	13		
Atlas	At	Philippines	45.8	4.7	2	6.9					1	7		
Bayugo	By	Philippines	-	-	-	0.82	0.22					14	32	
Boyongan	Bn	Philippines	67.4	-	1	1.4	0.22	-			1	14	32	
Tampakan	Tk	Philippines	85.0	-	1	7.7			4.09	0.14	1	7		44
Santo Nino	SN	Philippines	135.0	-	1	0.6					1	6		
Aksug	Ak	Russia (Siberia)	153.5	5.1	2	4.2					1	15		

Table S1.1, continued

Deposit	Acronym	Province	Sr/Y average	1σ	N. of Sr/Y values	Cu (Mt)	Overall duration of ore period (Ma)	1σ (Ma)	Magma cycle minimum duration (Ma)	1σ (Ma)	Ref. Sr/Y	Ref. Cu Mt	Ref. ore duration	ref. magma cycle duration
Zlatno	Zt	Slovakia	36.8	2.0	3	0.2					1	6		
Yulong	Yu	Tibet (China)	71.0	3.9	4	6.2					1	16		
Mangzong	Mz	Tibet (China)	81.6	-	1	0.2					1	16		
Silver Bell	SB	US (Arizona)	41.0	0.0	2	1.8					1	17		
Cu Creek	CCk	US (Arizona)	55.6	3.3	2	0.6					1	6		
Bagdad	Bg	US (Arizona)	59.8	31.4	3	6.4					1	18		
Ray	Ry	US (Arizona)	80.0	13.3	4	10.8					1	7		
Cu Basin	CBs	US (Nevada)	87.4	0.9	2	0.2					1	6		
Yerington	Ye	US (Nevada)	100.7	3.5	2	3.5					1	19		
Chino/Sta Rita	SR	US (N. Mexico)	35.9	8.1	2	8.7					1	20		
Bingham	Bh	US (Utah)	-	-	-	28.5	0.32	-	2			7	33	33
Butte	Bt	US (Montana)	-	-	-	35.1	1.5	-				7	34	
Bajo de la Alumbrera	BjA	Argentina	-	-	-	2.87	0.021	0.028				7	45	
Reko Diq	RD	Pakistan	56.8	12.3	52	24.2	0.7	0.064			46	47	47	

References

1. R.R. Loucks, Chemical characteristics, geodynamic settings, and petrogenesis of copper ore-forming arc magmas. *Centre for Exploration Targeting, CET Newsletter* (March 2012).
2. P. Schutte, M. Chiaradia, B. Beate, Petrogenetic Evolution of Arc Magmatism Associated with Late Oligocene to Late Miocene Porphyry-Related Ore Deposits in Ecuador. *Econ. Geol.* **105**, 1243-1270 (2010).
3. S. Asadi, F. Moore, A. Zarasvandi, Discriminating productive and barren porphyry copper deposits in the southeastern part of the central Iranian volcano-plutonic belt, Kerman region, Iran: A review. *Earth-Science Reviews* **138**, 25-46 (2014).
4. C. Chelle-Michou, M. Chiaradia, A. Ulianov, P. Beguelin, Petrologic evolution of the magmatic suite associated with the Coroccohuayco Cu(-Au-Fe) porphyry-skarn deposit, Peru. *J. Pet* (in press) (2015).
5. http://www.portergeo.com.au/database/mineinfo.asp?mineid=mn633
6. USGS "Porphyry copper deposits of the world" available at http://mrdata.usgs.gov/porcu/
7. D. Cooke, P. Hollings, J. L. Walshe, Giant porphyry deposits: Characteristics, distribution, and tectonic controls. *Econ. Geol.* **100**, 801–818 (2005).
8. http://www.portergeo.com.au/database/mineinfo.asp?mineid=mn1432
9. http://www.portergeo.com.au/database/mineinfo.asp?mineid=mn1427
10. http://www.portergeo.com.au/database/mineinfo.asp?mineid=mn620
11. http://www.portergeo.com.au/database/mineinfo.asp?mineid=mn1326
12. http://www.portergeo.com.au/database/mineinfo.asp?mineid=mn1270
13. http://www.portergeo.com.au/database/mineinfo.asp?mineid=mn940
14. http://www.portergeo.com.au/database/mineinfo.asp?mineid=mn882
15. http://www.portergeo.com.au/database/mineinfo.asp?mineid=mn972

16. http://www.portergeo.com.au/database/mineinfo.asp?mineid=mn428
17. http://www.portergeo.com.au/database/mineinfo.asp?mineid=mn210
18. http://www.portergeo.com.au/database/mineinfo.asp?mineid=mn881
19. http://www.portergeo.com.au/database/mineinfo.asp?mineid=mn393
20. http://www.portergeo.com.au/database/mineinfo.asp?mineid=mn436
21. K. J. Correa, O. M. Rabbia, L. B. Hernández, D. Selby, M. Astengo, The timing of magmatism and ore formation in the El Abra porphyry copper deposit, northern Chile: Implications for long-lived multiple-event magmatic-hydrothermal porphyry systems. *Econ. Geol.* (accepted pending revision).
22. V. Maksaev *et al.*, in *Andean metallogeny: new discoveries, concepts and updates*, R. H. Sillitoe, J. Perello, C. E. Vidal, Eds. (Society of Economic Geologists , Special Publication 11, Boulder, 2004), pp. 15–54.
23. J. Cannell, D.R. Cooke, J.L. Walshe, H. Stein, Geology, mineralization, alteration and structural evolution of the El Teniente porphyry Cu–Mo deposit. *Econ. Geol.* **100**, 979–1003 (2005).
24. K. Deckart, A. H. Clark, P. Cuadra, M. Fanning, Refinement of the time-space evolution of the giant Mio-Pliocene Río Blanco-Los Bronces porphyry Cu-Mo cluster, Central Chile: New U-Pb (SHRIMP II) and Re-Os geochronology and $^{40}Ar/^{39}Ar$ thermochronology data. *Mineralium Deposita* **48**, 57–79 (2013).
25. H. Stein, R. Markey, R. Sillitoe, J. Perello, Defining the lifespan of a Giant Porphyry Cu Deposit: Re-Os Dating at Los Pelambres, Chile. Geochim. Cosmochim. Acta **66-Supplement 1**, 738 (2002).
26. B. Romero *et al.*, Molybdenite Mineralization and Re-Os Geochronology of the Escondida and Escondida Norte Porphyry Deposits, Northern Chile. *Resource Geology* **61**, 91–100 (2011).
27. F. Barra *et al.*, Timing and formation of porphyry Cu–Mo mineralization in the Chuquicamata district, northern Chile: new constraints from the Toki cluster. *Mineralium Deposita* **48**, 629–651 (2013).
28. A. Zimmermann, H.J. Stein, J.W. Morgan, R.J. Markey, Y. Watanabe, Re–Os geochronology of the El Salvador porphyry Cu–Mo deposit, Chile: Tracking analytical improvements in accuracy and precision over the past decade. *Geochim. Cosmochim. Acta* **131**, 13-32 (2014).
29. P. Schutte, M. Chiaradia, F. Barra, D. Villagomez Diaz, B. Beate, Metallogenic features of Miocene porphyry Cu and porphyry-related mineral deposits in Ecuador revealed by Re-Os, $^{40}Ar/^{39}Ar$, and U-Pb geochronology. *Mineralium Deposita* **47**, 383-410 (2012).
30. S. Garwin, The geologic setting of intrusion-related hydrothermal systems near the Batu Hijau porphyry copper-gold deposit, Sumbawa, Indonesia. *Society of Economic Geologists Special Publication* **9**, 333–366 (2002).
31. C. Chelle-Michou, M. Chiaradia, D. Selby, M. Ovtcharova, R. A. Spikings, High-Resolution Geochronology of the Coroccohuayco Porphyry-Skarn Deposit, Peru: A Rapid Product of the Incaic Orogeny. *Econ. Geol.* **110**, 423-443 (2015).
32. D. P. Braxton, D. R. Cooke, From crucible to graben in 2.3 Ma: A high-resolution geochronological study of porphyry life cycles, Boyongan-Bayugo copper-gold deposits, Philippines. *Geology* **40**, 471–47 (2014).
33. A. Von Quadt, M. Erni, K. Martinek, M.Moll, I. Peytcheva, C. A. Heinrich, Zircon crystallization and the lifetimes of ore-forming magmatic-hydrothermal systems. *Geology* **39**, 731–734 (2011).
34. J. H. Dilles, H. J. Stein, M. W. Martin, Re-Os and U-Pb ages for the duration of the giant Butte, Montana, porphyry Cu-Mo and Cordilleran base metal lode ore deposit, paper presented at IAVCEI General Assembly 2004, Pucon, Chile (2004).
35. C.R. Stern, M.A. Skewes, A. Arévalo, Magmatic Evolution of the Giant El Teniente Cu–Mo Deposit, Central Chile. *J. Pet.* **52**, 1591–1617 (2011).
36. J. Perelló, R. H. Sillitoe, C. Mpodozis, H. Brockway, H. Posso, Geologic setting and evolution of the Porphyry copper-molybdenum and copper-gold deposits at Los Pelambres, Central Chile. *Society of Economic Geologists Special Publication* **16**, 79–104 (2012).
37. R. H. Sillitoe, J. K. Mortensen, Longevity of porphyry copper formation at Quellaveco, Peru. *Econ. Geol.* **105**, 1157–1162 (2010).
38. R. A. Padilla-Garza, S. R. Titley, C. J. Eastoe, Hypogene evolution of the Escondida porphyry copper deposit, Chile. *Society of Economic Geologists Special Publication* **11**, 141–165 (2004).
39. J. R. Ballard, J. M. Palin, I. S. Williams, I. H. Campbell, Two ages of porphyry intrusion resolved for the super-giant Chuquicamata copper deposit of northern Chile by ELA-ICP-MS and SHRIMP. *Geology* **29**, 383–386 (2001).
40. P. Cornejo *et al.*, El Salvador, Chile porphyry copper deposit revisited: geologic and geochronologic framework. *Internat. Geol. Rev.* **39**, 22-54 (1997).

41. P. Schutte, M. Chiaradia, B. Beate, Geodynamic controls on Tertiary arc magmatism in Ecuador: Constraints from U–Pb zircon geochronology of Oligocene–Miocene intrusions and regional age distribution trends. *Tectonophysics* **489**, 159-176 (2010).
42. C. Chelle-Michou, M. Chiaradia, M. Ovtcharova, A. Ulianov, J. F. Wotzlaw, Zircon petrochronology reveals the temporal link between porphyry systems and the magmatic evolution of their hidden plutonic roots (The Eocene Coroccohuayco deposit, Peru). *Lithos* **198-199**, 129-140 (2014).
43. B. Jones, V. Kamenetsky, P. Davidson, C. Allen, Antapaccay porphyry Cu-Au deposit: A product of Andean tectonism and evolving magmatism, late Eocene-early Oligocene Abancay batholith, Altiplano, southern Peru. Arizona Geological Society, Ores and Orogenesis: A Symposium Honoring the Career of William R. Dickinson, Tucson, Arizona, 2007, Program with Abstracts, pp. 132–133 (2007).
44. B. D. Rohrlach, R. R. Loucks, Multi-Million-Year Cyclic Ramp-Up of Volatiles in a Lower Crustal Magma Reservoir Trapped Below the Tampakan Cu-Au Deposit by Mio-Pliocene Crustal Compression in the Southern Philippines, in *Super Porphyry Copper & Gold Deposits - A Global Perspective*, T. M. Porter, Ed. (PGC Publishing, Adelaide, 2005), vol. 2, pp. 369–407.
45. Buret, Y. et al. From a long-lived upper-crustal magma chamber to rapid porphyry copper emplacement: Reading the geochemistry of zircon crystals at Bajo de la Alumbrera (NW Argentina). *Earth Planet. Sci. Lett.* **450**, 120-131.
46. Razique, A. magmatic evolution and genesis of the giant Reko Diq H14-H15 porphyry copper-gold deposit, district Chagai, Balochistan-Pakistan. PhD thesis, University of British Columbia, Vancouver, Canada, pp. 308 (2013).
47. Razique, A., Tosdal, R. M. & Creaser, R. A. Temporal evolution of the western porphyry Cu-Au systems at Reko Diq, Balochistan, western Pakistan. *Econ. Geol.* 109, 2003-2021 (2014).
