# Supplementary material for: Stochastic modelling of deep magmatic controls on porphyry copper deposit endowment
Source: Sci Rep. 2017 Mar 15;7:44523. doi: 10.1038/srep44523 (PMC5353633; doi:10.1038/srep44523)
Supplement: Supplementary Information 2 [file srep44523-s2.pdf]

Supplementary Information 2

Figures S2.1, S2.2, S2.3, S2.4, S2.5, S2.6, S2.7, Table S2.1 and modeling of Sr/Y values of the hybrid melts

Stochastic modelling of deep magmatic controls on porphyry copper deposit endowment

Massimo Chiaradia*, Luca Caricchi

Department of Earth Sciences, University of Geneva, Rue des Maraîchers 13, 1205 Geneva, Switzerland

**Corresponding author: Tel.: +41 22 379 66 34; Fax: +41 22 379 32 10; e-mail: Massimo.Chiaradia@unige.ch*

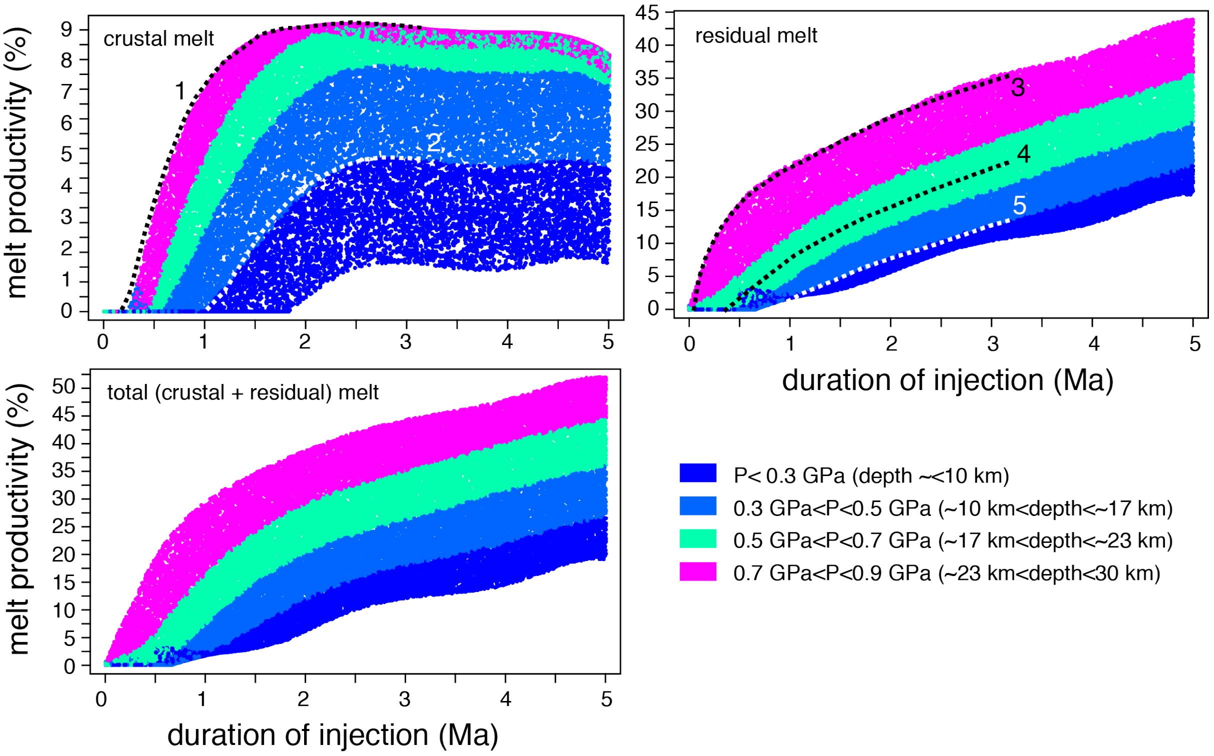

Figure S2.1: Results of the parameterization of melt productivities for crustal and residual melts from the model of Annen et al. (2006)[1] and their Monte Carlo simulations. Dashed curves represent the curves of the model of Annen et al. (2006) [1] and the coloured dots represent the results of our parametrization and ~100000 Monte Carlo simulations. Dashed curves labels: 1. crustal melt productivity from the model of Annen et al. (2006) [1] at depth of 30 km from partial melting of amphibolitic crust; 2. crustal melt productivity from the model of Annen et al. (2006)[1] at depth of 10 km from partial melting of graywacke crust; 3., 4., 5. residual melt productivities from the model of Annen et al. (2006) [1] for basalt fractionation at depths of 30 km, 20 and 10 km respectively. We have extrapolated the results of the model of Annen et al. (2006) [1] to higher durations of basaltic injections (5 Ma) and to slightly lower pressures (0.15 GPa).

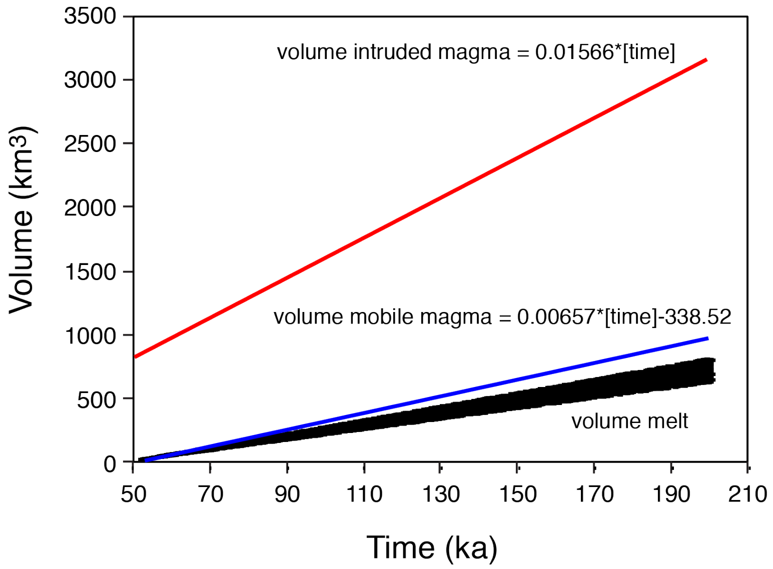

Figure S2.2: Volume of intruded magma and of mobile magma from Annen et al. (2009) [2]. Volume of melt is calculated from volume of mobile magma assuming that the melt fraction in the mobile magma ranges randomly between 60 and 80% of the magma volume (Annen et al., 2009)[2].

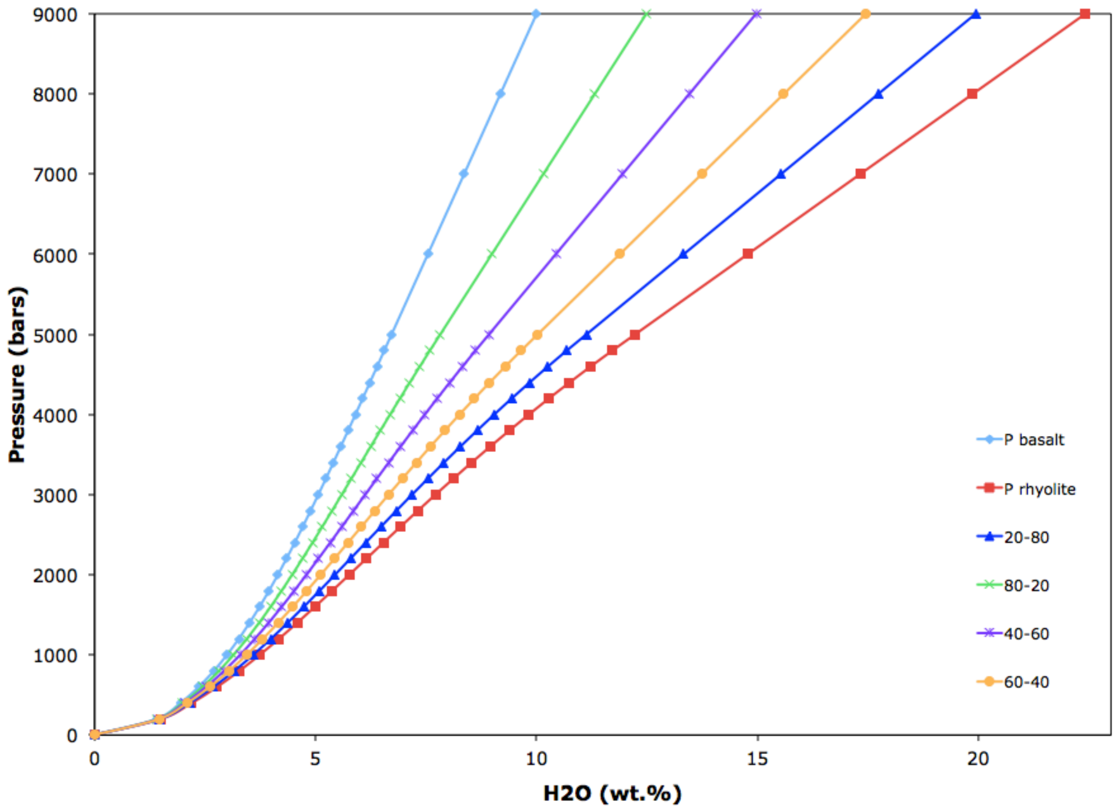

Figure S2.3: H_2O solubility relationship to pressure and melt composition calculated from VolatileCalc[3]. Solubilities for intermediate compositions are interpolated (e.g., 20-80 means 20% of basalt and 80% of rhyolite, etc.). Solubilities above 0.5 GPa up to 0.9 GPa are extrapolated.

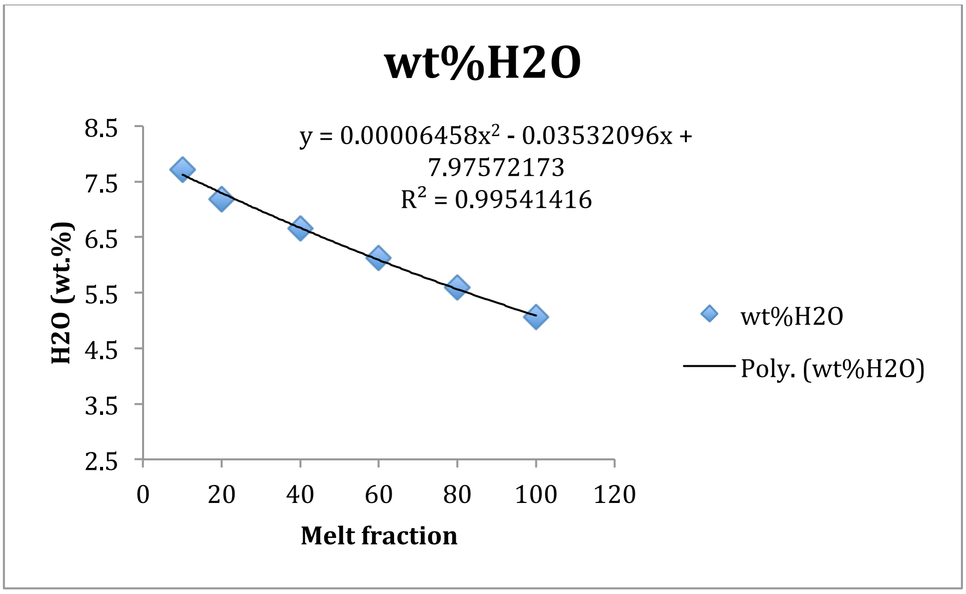

Figure S2.4: H_2O solubility is linked to melt fraction (M) by a best-fit polynomial equation.

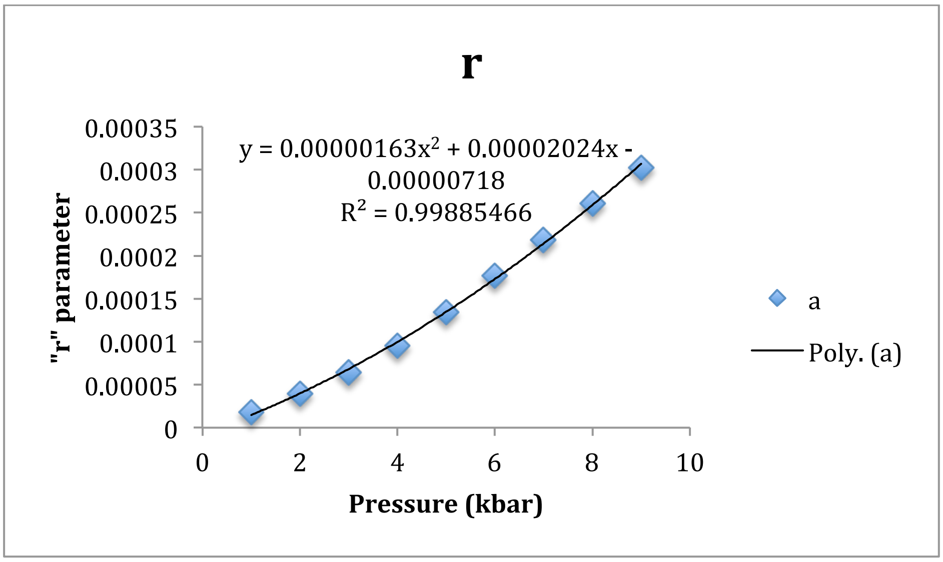

Figure S2.5: Pressure dependence of the parameter r (similar dependencies exist also for the parameters s and t) in the 2nd order polynomial equation relating the H_2O concentration to melt fraction.

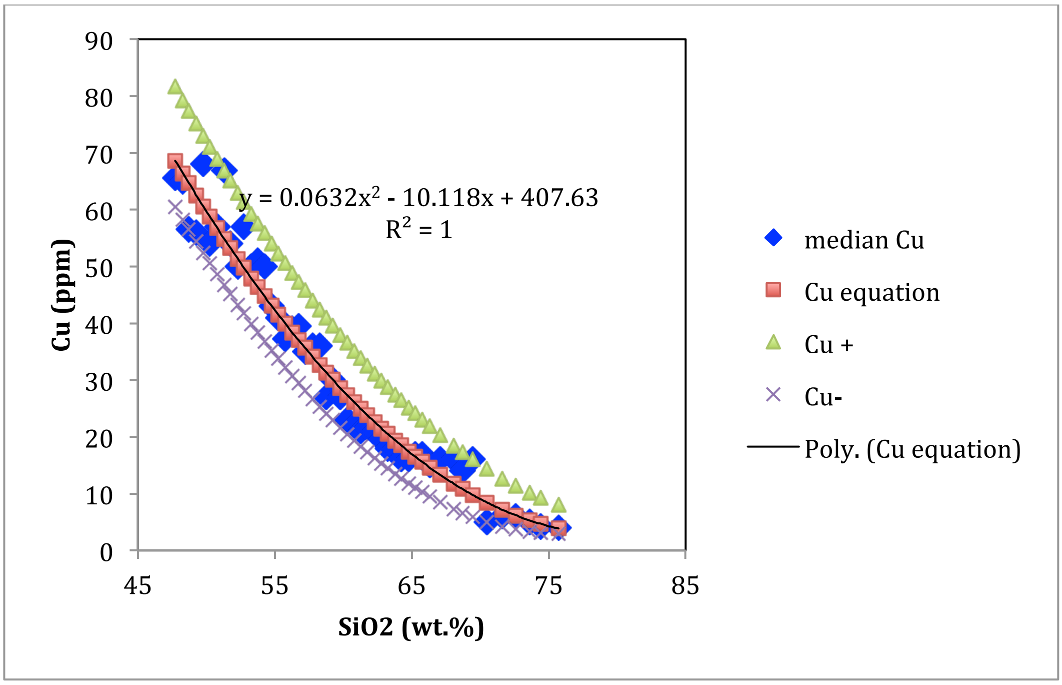

Figure S2.6: Median values of copper contents of whole magmatic rocks of thick arcs steadily decrease with SiO_2 (from Chiaradia, 2014)[4]. The upper (Cu+) and lower (Cu-) curves encompass the range of variability of the median values. We randomly range values of Cu contents within these curves to obtain the Cu contents in fluids exsolved from magmas with specific SiO_2 contents using random fluid-melt partition coefficients for Cu between 2 and 100.

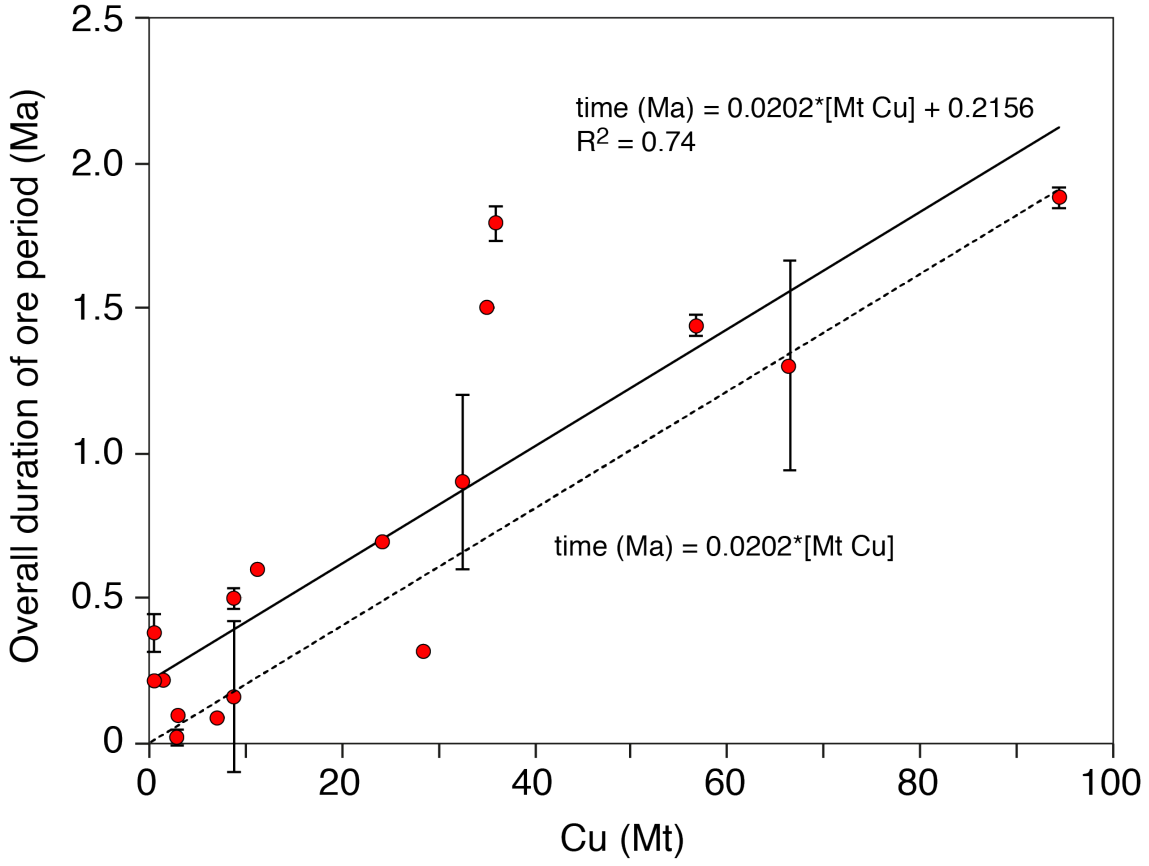

Figure S2.7: Linear relationship between ore duration and Cu (Mt) endowment for 17 porphyry copper deposits (red circles; Table S1) and the equation expressing the best fit linear regression constrained to pass through the origin of the diagram. Uncertainty bars are 1σ for overall duration of ore period (see Table S1).

Table S2.1: Relationship between lithology, SiO_2 content and melt fraction.

Lithology	*SiO_2 (mid-value of TAS diagram5)*	*Melt Fraction (M) (mid-value from Annen et al., 2006)[1]*
Basalt	76	0.1
Basaltic andesite	70	0.2
Andesite	63	0.34
Dacite	57	0.5
Rhyolite	49	1

Modelling of Sr/Y values of the hybrid melts

a. We have collected available partition coefficients (KDs) from GERM (https://earthref.org/KDD/) for Sr and Y between various minerals and melts of changing compositions (spreadsheets "Sr KDs" and "Y KDs" of Supplementary Dataset 1). GERM provides KDs for minerals in equilibrium with melts of different generic compositions (i.e., basalt, basaltic andesite, andesite, dacite, rhyolite). Based on the Table S1.1 above, we have attributed to each lithology an SiO_2 value and converted this into residual melt fraction, assuming that these melts were derived from fractional crystallization of basalt. The pool of Sr and Y KD values for each specific lithology was used to obtain a median value for that lithology (corresponding to a certain melt fraction). The so obtained median values of Sr KDs display systematic increases with

decreasing melt fraction that can be best fitted with power or polynomial equations (see graphs in spreadsheets "Sr KDs" and "Y KDs" of Supplementary Dataset 1).

b. We have gathered experimental results of mineral assemblages and their proportions forming from crystallization of hydrous basaltic melts at various T and P available from the literature[6,7]. Percentages of the minerals (glass = residual melt in the experiments) were recalculated to 100% to obtain the fractionating mineral assemblage. For the experiments of Müntener et al. (2001)[6] we have retained only those with initial water content of 3.8 wt.% which best approximates the interval of initial H_2O content in the basalt of our model (2-4 wt.%). Using the above Sr and Y KDs (which systematically change with residual melt fraction) we have calculated the resulting Sr and Y bulk KDs for the different residual melt fractions corresponding to experiments carried out at different P and T. Then we have calculated the Sr and Y values of the residual melt for each run assuming initial Sr and Y contents of 500 and 20 ppm (typical of arc basalts: Kelemen et al., 2004)[8]. The resulting Sr/Y values of the residual melt define three distinct P-dependent trends with respect to residual melt fraction (=glass in the experiments), ranging from decreasing Sr/Y with decreasing melt fraction at low pressure to increasing Sr/Y with decreasing melt fraction at higher pressures ("Fractionating assemblages" spreadsheet in Supplementary Dataset 1).

c. The three trends of Sr/Y versus melt fraction identified for different median values of Pressure (0.1, 0.26 and 1.2 GPa) at which experiments were carried out ("Fractionating assemblages" spreadsheet in Supplementary Dataset 1) were fitted with 3rd and 2nd order polynomial equations. The parameters a, b, c, d of the polynomial equations (y= ax^3, + bx^2, + cx + d) were then fitted through 2nd order polynomial equations to obtain their variations with pressure. Ultimately we obtain by this way a system of equations that allow us to calculate Sr/Y values of residual melts for any melt fraction and at any pressure between ca. 0.1 and 1.2 GPa. We input this in our Monte Carlo simulations obtaining Sr/Y of the melts formed at various depths and after different injection durations according to the model of Annen et al. (2006)[1].

References

[1]Annen, C., Blundy, J. D., & Sparks, R. S. J. The genesis of intermediate and silicic magmas in deep crustal hot zones. *J. Pet.* **47**, 505–539 (2006).

[2]Annen, C. From plutons to magma chambers: Thermal constraints on the accumulation of eruptible silicic magma in the upper crust. *Earth Planet. Sci. Lett.* **284**, 409–416 (2009).

[3]Newman, S. & Lowenstern, J. B. VolatileCalc: a silicate melt-H_2O-CO_2 solution model written in Visual Basic for Excel. *Computers and Geosciences* **28**, 597-604 (2002).

[4]Chiaradia M., Copper enrichment in arc magmas controlled by over- riding plate thickness. *Nature Geoscience* **7**, 43–46 (2014).

[5]Le Bas, M.J., Le Maitre, R.W., Streckeisen, A., & Zanettin, B. A chemical classification of volcanic rocks based on the total alkali-silica diagram. *J. Pet.* **27**, 745-750 (1986).

[6]Moore G. & Carmichael, L. S. E. The hydrous phase equilibria (to 3 kbar) of an andesite and basaltic andesite from western Mexico: constraints on water content and conditions of phenocryst growth. *Contrib. Mineral. Petrol.* **130**, 304-319 (1998).

[7]Müntener O., Kelemen, P. O., & Grove, T. L. The role of H_2O during crystallization of primitive arc magmas under uppermost mantle conditions and genesis of igneous pyroxenites: an experimental study. *Contrib. Mineral. Petrol.* **141**, 643-658 (2001).

[8]Kelemen, P.B., Hanghoj, K., & Greene, A.R. One View of the Geochemistry of Subduction-related Magmatic Arcs, with an Emphasis on Primitive Andesite and Lower Crust. In: Holland, H.D. & Turekian, K.K. (Eds.), *Treatise on Geochemistry*, Elsevier, Amsterdam, v. 3, pp. 593-659 (2004).
