# Supplementary material for: Stochastic modelling of deep magmatic controls on porphyry copper deposit endowment
Source: Sci Rep. 2017 Mar 15;7:44523. doi: 10.1038/srep44523 (PMC5353633; doi:10.1038/srep44523)
Supplement: Supplementary Information 3 [file srep44523-s3.pdf]

Supplementary Information 3

Tables S3.1, S3.2

Stochastic modelling of deep magmatic controls on porphyry copper deposit endowment

Massimo Chiaradia*, Luca Caricchi

Department of Earth Sciences, University of Geneva, Rue des Maraîchers 13, 1205 Geneva, Switzerland

**Corresponding author: Tel.: +41 22 379 66 34; Fax: +41 22 379 32 10; e-mail: Massimo.Chiaradia@unige.ch*

Table S3.1: Description of all parameters used in simulations for melt injection rate of 5 mm/a through a disk of 7500 m radius (equivalent to 0.0009 km^3/a) (Supplementary Dataset 2).

A	Time = time since first injection starts (random between 0 and 5 Ma)
B	H_2O parent = wt.% content of H_2O in parent magma (random between 2 and 4 wt.%)
C	H_2O assimilant = wt.% content of H_2O in assimilant (random between 0.5 and 1 wt.%)
D	P (kbars) = pressure at which magma accumulation occurs (random between 1.5 and 9 kbars)
E	a Pressure = a parameter of the fifth order equation ($y=ax^5+bx^4 +cx^3 dx^2 + ex + f$) describing the total melt fraction as a function of P of saturation
F	b Pressure = b parameter of the second order equation ($y=ax^2+bx +c$) describing the total melt fraction as a function of P of saturation
G	c Pressure = c parameter of the second order equation ($y=ax^2+bx +c$) describing the total melt fraction as a function of P of saturation
H	P saturation = pressure at which magma is H_2O-saturated as a function of the second order polynomial equation P saturation = [a Pressure]*[H_2O in melt]^2 + [b Pressure]*[H2O in melt] + [c Pressure]
I	Delta P = P of magma formation minus pressure of H_2O saturation for that magma
J	H_2O in melt = wt.% content of H_2O in the melt
K	a crustal = a parameter of the second order equation ($y=ax^2+bx +c$) describing the dependence on time of crustal melt fraction produced at a random depth from parameterization of Annen et al. (2006)[1].
L	b crustal = b parameter of the second order equation ($y=ax^2+bx +c$) describing the dependence on time of crustal melt fraction produced at a certain depth from parameterization of Annen et al. (2006)[1].
M	c crustal = c parameter of the second order equation ($y=ax^2+bx +c$) describing the dependence on time of crustal melt fraction produced at a certain depth from parameterization of Annen et al. (2006)[1].
N	Crustal melt fraction Annen = crustal melt fraction parameterized from Annen et al. (2006)[1] model.
O	Crustal melt fraction Annen logical test = logical test for crustal melt fraction calculated from Annen et al. (2006)[1] model: if the parameterized value is <0 → 0, otherwise is the parameterized value.
P	Crustal melt fraction Annen logical test 2 = logical test 2 for crustal melt fraction calculated from Annen et al. (2006)[1] model to avoid negative values below 1 Ma.
Q	Crustal melt fraction Annen logical test 3 = logical test 3 for crustal melt fraction calculated from Annen et al. (2006)[1] model to avoid negative values below 0.25 Ma.
R	Magma rate = fixed magma rate at 5 mm/a (the magma rate used by Annen et al. (2006)[1] for the parameterization of the crustal and residual melt fractions produced at different depths).
S	Magma rate time integrated = magma rate integrated over the random time of the same row.
T	Disk radius = fixed radius of 7500 m for a circular shape section through which the above flux rate is integrated. This radius is typical of crustal batholiths (Annen, 2009)[2]. The integrated magma volume corresponds to 0.0009 km^3/a, which is a typical average long-term flux for arc magmatism.
U	Volume of crustal melt integrated over time = volume of crustal melt produced by basaltic melt passing through a circle with radius equal to 7500 m at the depth value (pressure) for the same row.
V	Tons of crustal melt time integrated = the above volume multiplied by an average andesitic melt density of 2.6 gm/cm^3. (Although density changes with melt composition we have assumed for simplicity an average andesitic melt density for melts ranging from basaltic to rhyolitic).
W	Crustal melt% Annen = % of crustal melt fraction from parameterization of Annen et al. (2006)[1]
X	a residual = a parameter of the second order equation ($y=ax^2+bx +c$) describing the dependence on time of residual melt fraction produced at a random depth from parameterization of Annen et al. (2006)[1].

Y	b residual = b parameter of the second order equation ($y=ax^2+bx+c$) describing the dependence on time of residual melt fraction produced at a random depth from parameterization of Annen et al. (2006)[1].
Z	c residual = c parameter of the second order equation ($y=ax^2+bx+c$) describing the dependence on time of residual melt fraction produced at a random depth from parameterization of Annen et al. (2006)[1].
AA	residual Melt fraction Annen = residual melt fraction parameterized from Annen et al. (2006)[1] model
AB	residual Melt fraction Annen logical test = logical test for residual melt fraction parameterized from Annen et al. (2006)[1] model to avoid negative values below 0.75 Ma.
AC	residual Melt fraction Annen logical test2 = logical test 2 for residual melt fraction parameterized from Annen et al. (2006)[1] model to avoid negative values below 0.5 Ma.
AD	Magma rate (tons/Ma/km arc) = fixed magma rate at 5 mm/a (the magma rate used by Annen et al., 2006[1], for the parameterization of the crustal and residual melt fractions produced at different depths).
AE	V residual melt fraction time integrated = volume of residual melt produced by basaltic melt passing through a circle with radius equal to 7500 m at the depth value (pressure) for the same row.
AF	Magma rate time integrated (tons/Ma/km arc) = magma rate integrated over the random time of the same row
AG	tons residual melt fraction time integrated = the above volume multiplied by an average andesitic melt density of 2.6 gm/cm^3. (Although density changes with melt composition we have assumed for simplicity an average andesitic melt density for melts ranging from basaltic to rhyolitic).
AH	SiO_2 total = SiO_2 relationship to melt fraction (F) based on the empirical equation [$SiO_2 = 35.436F^2 - 68.859F + 82.439$]
AI	all melt % = sum of residual and crustal melts percentages
AJ	residual Melt% Annen = % of residual melt parameterized from Annen et al. (2006)[1]
AK	H_2O wt% in residual melt = H_2O wt.% concentration in the residual melt from random initial concentration (H_2O_{in}=2-4 wt.%) assuming a completely incompatible behavior for H_2O during magma fractionation ($=100*H_2O_{in}*F^{-1}$, where F is the residual melt fraction)
AL	log test H_2O wt% in residual melt = H_2O wt.% in residual melt assuming that the minimum residual melt remaining after fractionation is 10%
AM	a H_2O = a parameter of the second order equation ($y=ax^2+bx+c$) describing the dependence on pressure of the H_2O content of a silicate melt of a random composition (=melt fraction) paramaterized from the P-X dependency of H_2O solubility of VolataileCalc (Newman and Lowenstern, 2002)[3]
AN	b H_2O = b parameter of the second order equation ($y=ax^2+bx+c$) describing the dependence on pressure of the H_2O content of a silicate melt of a random composition (=melt fraction) paramaterized from the P-X dependency of H_2O solubility of VolataileCalc (Newman and Lowenstern, 2002)[3]
AO	c H_2O = c parameter of the second order equation ($y=ax^2+bx+c$) describing the dependence on pressure of the H_2O content of a silicate melt of a random composition (=melt fraction) paramaterized from the P-X dependency of H_2O solubility of VolataileCalc (Newman and Lowenstern, 2002)[3]
AP	residual melt Annen logical test for H_2O solubility (F=100-10%) = = logical test for residual melt fraction parameterized from Annen et al. (2006)[1] model to avoid residual melt fractions <10%
AQ	H_2O solubility in melt (F =100-10 %) = H_2O solubility in the residual melt at the random pressure and residual melt fraction (logical test corrected) of the same raw
AR	H_2O wt% in residual melt logical test 100-10% F range = effective H_2O wt% content of the residual melt corrected for oversaturation (i.e., if the theoretical amount of H_2O in the residual melt is higher than the maximum permissible H_2O content in that melt composition and at that specific P, then the latter value is taken as the effective H_2O content of that simulation and the remainder H_2O is in excess and must have been exsolved).

AS	Excess residual H_2O = H_2O in excess according to the above explanation
AT	H_2O wt% in crust = wt.% content of H_2O in assimilant (random between 0.2 and 1 wt.%)
AU	H_2O wt% in partial melt = H_2O wt.% concentration in the crustal melt from random initial concentration (H_2O_{in}=0.2-1 wt.%) assuming a completely incompatible behavior for H_2O during partial melting (=100*H_2O_{in}*F^{-1}, where F is the crustal melt fraction)
AV	H_2O solubility (wt.%) with Annen crustal melt % = H_2O solubility in the crustal melt at the random pressure and crustal melt fraction (logical test corrected) of the same raw
AW	H_2O solubility (wt.%) with Annen crustal melt % logical test = effective H_2O wt% content of the crustal melt corrected for oversaturation (i.e., if the theoretical amount of H_2O in the crustal melt is higher than the maximum permissible H_2O content in that melt composition and at that specific P, then the latter value is taken as the effective H_2O content of that simulation and the remainder H_2O is in excess and must have been exsolved).
AX	Excess H_2O partial melt = H_2O in excess according to the above explanation
AY	H_2O residual (tons) = tons of H_2O dissolved in residual melt
AZ	Log test excess residual H_2O tons = tons of excess H_2O (logical test to avoid negative values, if negative means that excess H_2O is 0)
BA	Excess H_2O residual (tons) = tons of excess H_2O from residual melt (H_2O that has been liberated by the residual melt at specific P, time and composition values occurring in the same raw)
BB	H_2O crustal (tons) = tons of H_2O dissolved in crustal melt
BC	Excess H_2O crustal (tons) = tons of H_2O liberated by the crustal melt at specific P, time and composition values occurring in the same raw
BD	log test excess crustal H_2O tons = tons of excess H_2O (logical test to avoid negative values, if negative means that excess H_2O is 0)
BE	H_2O total in melt (tons) = sum of tons of H_2O dissolved in the residual and crustal melt
BF	Excess H_2O total in melt (tons) = sum of the tons of excess H_2O liberated by both the crustal and residual melt
BG	Excess total H_2O NEW log test = sum of the tons of excess H_2O liberated by both the crustal and residual melt avoiding negative values (logical tests for excess H_2O in crustal and residual melts)
BH	M melt - excess H_20 tons = mass in tons of the total melt (residual + crustal) minus the sum of excess H_2O (residual + crustal)
BI	M total melt ton =total tons of residual + crustal melts
BJ	% H_2O excess = wt% of total excess H_2O
BK	M H_2O excess ton = BV
BL	Kd = Cu KD between melt and fluid random values between 2 and 100
BM	Cu total melt ppm = CZ
BN	Cu tot ton = tons of Cu in melt
BO	Cu ppm res melt = ppm of Cu in H_2O-saturated melt (where part of the Cu has been lost to the exsolved H_2O)
BP	Cu ppm fluid = ppm Cu in the exsolved fluid of the oversaturated melt
BQ	Cu tot res melt Mt = Mt of Cu in the “residual” oversaturated melt (this is identical to the total Cu amount of melt only in undersaturated melts)
BR	Cu tot in excess fluid Mt = Mt of Cu that are lost by the oversaturated melt to the exsolved fluid
BS	50% Cu tot in excess fluid Mt = 50% of Mt of Cu that are lost by the oversaturated melt to the exsolved fluid
BT	Cu tot Mt = Mt of Cu in melt
BU	M res hydrous melt ton = tons of residual hydrous melt (this is equivalent to the tons of undersaturated melt)
BV	exsolvable H_2O tons = tons of H_2O dissolved in residual hydrous melt
BW	Kd = BL
BX	Kd = Cu KD between melt and fluid random values between 2 and 100
BY	Cu total melt ppm = ppm of Cu in total melt
BZ	Cu tot ton = tons of Cu in hydrous under and oversaturated melt
CA	Cu res melt = ppm of Cu in the hydrous melt after it exsolved H_2O at the saturation P

CB	Cu fluid = ppm of Cu in the fluid exsolved at saturation depth
CC	Cu tot res melt Mt = Mt of Cu in the hydrous melt after H_2O exsolution
CD	Cu tot in exsolvable fluid Mt = Mt of Cu in exsolvable fluid at saturation depth
CE	Cu tot Mt = Mt of Cu total in hydrous melt (melt + exsolved fluid)
CF	50% Cu fluid Mt = 50% of T
CG	Total melt (tons) = tons of total melt (residual + crustal)
CH	a Cu = a parameter of the second order equation ($y=ax^2+bx+c$, where y = Cu (ppm) and x = SiO_2 (wt.%)) describing the Cu concentration variability at any SiO_2 value parameterized from the median values of Cu and SiO_2 from continental arc rocks (from Chiaradia, 2014)[4].
CI	b Cu = b parameter of the second order equation ($y=ax^2+bx+c$, where y = Cu (ppm) and x = SiO_2 (wt.%)) describing the Cu concentration variability at any SiO_2 value parameterized from the median values of Cu and SiO_2 from continental arc rocks (from Chiaradia, 2014)[4].
CJ	c Cu = c parameter of the second order equation ($y=ax^2+bx+c$, where y = Cu (ppm) and x = SiO_2 (wt.%)) describing the Cu concentration variability at any SiO_2 value parameterized from the median values of Cu and SiO_2 from continental arc rocks (from Chiaradia, 2014)[4].
CK	Cu ppm = Cu ppm of melt
CL	Cu ppm melt = CZ
CM	Molar V H_2O = molar volume of H_2O at the random pressure of the corresponding row. This has been calculated
CN	H_2O% in hydrous melt= wt% of H_2O in over or undersaturated hydrous melt
CO	Moles of excess H_2O = moles of H_2O exsolved from oversaturated melts
CP	Volume cc excess H_2O = cubic centimeters of H_2O exsolved from oversaturated melts
CQ	Volume km^3 total melt = volume (km^3) of total melt
CR	Volume km^3 excess H_2O = volume (km^3) of H_2O exsolved from oversaturated melts
CS	Volume km^3 hydrous melt = volume (km^3) of hydrous melt
CT	Volume % excess H_2O = volume % of H_2O exsolved from oversaturated melts
CU	Ore Duration avg (Ma) = duration of the ore event based on the transfer of the average values of the magma volumes produced at the average transfer rate
CV	Cu flux exsolvable tons/a = Total of exsolvable Cu in undersaturated melts divided by the average ore duration
CW	50% Cu flux = same as above but divided by 2
CX	aa- = minimum value of the "aa parameter" describing the second order polynomial pressure dependency of the "aa parameter" (=a eq below; a eq = aa*pressure^2 + ab*pressure + ac) in the third order polynomial equation describing the variation of Sr/Y values with melt fraction
CY	aa+ = maximum value of the "aa parameter" describing the second order polynomial pressure dependency of the "a parameter" (=a eq below; a eq = aa*pressure^2 + ab*pressure + ac) in the third order polynomial equation describing the variation of Sr/Y values with melt fraction
CZ	aa = random value of the "aa parameter" between minimum and maximum values above describing the pressure dependency of the "a parameter" (=a eq below; a eq = aa*pressure^2 + ab*pressure + ac) in the third order polynomial equation describing the variation of Sr/Y values with melt fraction
DA	ab- = minimum value of the "ab parameter" describing the second order polynomial pressure dependency of the "a parameter" (=ab eq below; a eq = aa*pressure^2 + ab*pressure + ac) in the third order polynomial equation describing the variation of Sr/Y values with melt fraction
DB	ab+ = maximum value of the "ab parameter" describing the second order polynomial pressure dependency of the "a parameter" (=a eq below; a eq = aa*pressure^2 + ab*pressure + ac) in the third order polynomial equation describing the variation of Sr/Y values with melt fraction
DC	ab = random value of the "ab parameter" between minimum and maximum values above describing the pressure dependency of the "a parameter" (=a eq below; a eq = aa*pressure^2 + ab*pressure + ac) in the third order polynomial equation describing the variation of Sr/Y values with melt fraction

DD	ac- = minimum value of the "ac parameter" describing the second order polynomial pressure dependency of the "a parameter" (=a eq below; a eq = aa*pressure^2 + ab*pressure + ac) in the third order polynomial equation describing the variation of Sr/Y values with melt fraction
DE	ac+ = maximum value of the "ac parameter" describing the second order polynomial pressure dependency of the "a parameter" (=a eq below; a eq = aa*pressure^2 + ab*pressure + ac) in the third order polynomial equation describing the variation of Sr/Y values with melt fraction
DF	ac = random value of the "ac parameter" between minimum and maximum values above describing the pressure dependency of the "a parameter" (=a eq below; a eq = aa*pressure^2 + ab*pressure + ac) in the third order polynomial equation describing the variation of Sr/Y values with melt fraction
DG	a eq = aa*pressure^2 + ab*pressure + ac; a eq is the pressure-dependent variation of the a parameter in the third order polynomial equation (Sr/Y = [a eq]*[melt fraction]^3 + [b eq]*[melt fraction]^2 + [c eq]*[melt fraction] + [d eq]) describing the variation of Sr/Y values with melt fraction
DH	ba- = minimum value of the "ba parameter" describing the second order polynomial pressure dependency of the "b parameter" (=b eq below; b eq = ba*pressure^2 + bb*pressure + bc) in the third order polynomial equation describing the variation of Sr/Y values with melt fraction
DI	ba+ = maximum value of the "ba parameter" describing the second order polynomial pressure dependency of the "b parameter" (=b eq below; b eq = ba*pressure^2 + bb*pressure + bc) in the third order polynomial equation describing the variation of Sr/Y values with melt fraction
DJ	ba = random value of the "ba parameter" between minimum and maximum values above describing the pressure dependency of the "b parameter" (=b eq below; b eq = ba*pressure^2 + bb*pressure + bc) in the third order polynomial equation describing the variation of Sr/Y values with melt fraction
DK	bb- = minimum value of the "bb parameter" describing the second order polynomial pressure dependency of the "b parameter" (=b eq below; b eq = ba*pressure^2 + bb*pressure + bc) in the third order polynomial equation describing the variation of Sr/Y values with melt fraction
DL	bb+ = maximum value of the "bb parameter" describing the second order polynomial pressure dependency of the "b parameter" (=c eq below; b eq = aa*pressure^2 + ab*pressure + ac) in the third order polynomial equation describing the variation of Sr/Y values with melt fraction
DM	bb = random value of the "bb parameter" between minimum and maximum values above describing the pressure dependency of the "b parameter" (=b eq below; b eq = aa*pressure^2 + ab*pressure + ac) in the third order polynomial equation describing the variation of Sr/Y values with melt fraction
DN	bc- = minimum value of the "bc parameter" describing the second order polynomial pressure dependency of the "b parameter" (=b eq below; b eq = aa*pressure^2 + ab*pressure + ac) in the third order polynomial equation describing the variation of Sr/Y values with melt fraction
DO	bc+ = maximum value of the "bc parameter" describing the second order polynomial pressure dependency of the "b parameter" (=b eq below; b eq = aa*pressure^2 + ab*pressure + ac) in the third order polynomial equation describing the variation of Sr/Y values with melt fraction
DP	bc = random value of the "bc parameter" between minimum and maximum values above describing the pressure dependency of the "b parameter" (=b eq below; b eq = aa*pressure^2 + ab*pressure + ac) in the third order polynomial equation describing the variation of Sr/Y values with melt fraction
DQ	b eq = ba*pressure^2 + bb*pressure + bc; b eq is the pressure-dependent variation of the b parameter in the third order polynomial equation (Sr/Y = [a eq]*[melt fraction]^3 + [b eq]*[melt fraction]^2 + [c eq]*[melt fraction] + [d eq]) describing the variation of Sr/Y values with melt fraction
DR	ca- = minimum value of the "ca parameter" describing the second order polynomial pressure dependency of the "c parameter" (=c eq below; c eq = ca*pressure^2 + cb*pressure + cc) in the third order polynomial equation describing the variation of

	Sr/Y values with melt fraction
DS	ca+ = maximum value of the “ca parameter” describing the second order polynomial pressure dependency of the “c parameter” (=c eq below; c eq = ca*pressure^2 + cb*pressure + cc) in the third order polynomial equation describing the variation of Sr/Y values with melt fraction
DT	ca = random value of the “ca parameter” between minimum and maximum values above describing the pressure dependency of the “c parameter” (=c eq below; c eq = ca*pressure^2 + cb*pressure + cc) in the third order polynomial equation describing the variation of Sr/Y values with melt fraction
DU	cb- = minimum value of the “cb parameter” describing the second order polynomial pressure dependency of the “c parameter” (=c eq below; c eq = ca*pressure^2 + cb*pressure + cc) in the third order polynomial equation describing the variation of Sr/Y values with melt fraction
DV	cb+ = maximum value of the “cb parameter” describing the second order polynomial pressure dependency of the “c parameter” (=c eq below; c eq = ca*pressure^2 + cb*pressure + cc) in the third order polynomial equation describing the variation of Sr/Y values with melt fraction
DW	cb = random value of the “cb parameter” between minimum and maximum values above describing the pressure dependency of the “c parameter” (=c eq below; c eq = ca*pressure^2 + cb*pressure + cc) in the third order polynomial equation describing the variation of Sr/Y values with melt fraction
DX	cc- = minimum value of the “cc parameter” describing the second order polynomial pressure dependency of the “c parameter” (=c eq below; c eq = ca*pressure^2 + cb*pressure + cc) in the third order polynomial equation describing the variation of Sr/Y values with melt fraction
DY	cc+ = maximum value of the “cc parameter” describing the second order polynomial pressure dependency of the “c parameter” (=c eq below; c eq = ca*pressure^2 + cb*pressure + cc) in the third order polynomial equation describing the variation of Sr/Y values with melt fraction
DZ	cc = random value of the “cc parameter” between minimum and maximum values above describing the pressure dependency of the “c parameter” (=c eq below; c eq = ca*pressure^2 + cb*pressure + cc) in the third order polynomial equation describing the variation of Sr/Y values with melt fraction
EA	c eq = ca*pressure^2 + cb*pressure + cc; c eq is the pressure-dependent variation of the a parameter in the third order polynomial equation (Sr/Y = [a eq]*[melt fraction]^3 + [b eq]*[melt fraction]^2 + [c eq]*[melt fraction] + [d eq]) describing the variation of Sr/Y values with melt fraction
EB	da- = minimum value of the “da parameter” describing the second order polynomial pressure dependency of the “d parameter” (=d eq below; d eq = da*pressure^2 + db*pressure + dc) in the third order polynomial equation describing the variation of Sr/Y values with melt fraction
EC	da+ = maximum value of the “da parameter” describing the second order polynomial pressure dependency of the “cd parameter” (=d eq below; d eq = da*pressure^2 + db*pressure + dc) in the third order polynomial equation describing the variation of Sr/Y values with melt fraction
ED	da = random value of the “da parameter” between minimum and maximum values above describing the pressure dependency of the “c parameter” (=d eq below; d eq = da*pressure^2 + db*pressure + dc) in the third order polynomial equation describing the variation of Sr/Y values with melt fraction
EE	db- = minimum value of the “db parameter” describing the second order polynomial pressure dependency of the “d parameter” (=d eq below; d eq = da*pressure^2 + db*pressure + dc) in the third order polynomial equation describing the variation of Sr/Y values with melt fraction
EF	db+ = maximum value of the “db parameter” describing the second order polynomial pressure dependency of the “d parameter” (=d eq below; d eq = da*pressure^2 + db*pressure + dc) in the third order polynomial equation describing the variation of Sr/Y values with melt fraction
EG	db = random value of the “db parameter” between minimum and maximum values above describing the pressure dependency of the “d parameter” (=d eq below; d eq =

	da*pressure^2 + db*pressure + dc) in the third order polynomial equation describing the variation of Sr/Y values with melt fraction
EH	dc- = minimum value of the "dc parameter" describing the second order polynomial pressure dependency of the "d parameter" (=d eq below; d eq = da*pressure^2 + db*pressure + dc) in the third order polynomial equation describing the variation of Sr/Y values with melt fraction
EI	dc+ = maximum value of the "dc parameter" describing the second order polynomial pressure dependency of the "d parameter" (=d eq below; d eq = da*pressure^2 + cdb*pressure + dc) in the third order polynomial equation describing the variation of Sr/Y values with melt fraction
EJ	dc = random value of the "dc parameter" between minimum and maximum values above describing the pressure dependency of the "d parameter" (=d eq below; d eq = da*pressure^2 + db*pressure + dc) in the third order polynomial equation describing the variation of Sr/Y values with melt fraction
EK	d eq = da*pressure^2 + db*pressure + dc; d eq is the pressure-dependent variation of the a parameter in the third order polynomial equation (Sr/Y = [a eq]*[melt fraction]^3 + [b eq]*[melt fraction]^2 + [c eq]*[melt fraction] + [d eq]) describing the variation of Sr/Y values with melt fraction
EL	Sr/Y = [a eq]*[melt fraction]^3 + [b eq]*[melt fraction]^2 + [c eq]*[melt fraction] + [d eq]) describes the variation of Sr/Y values with melt fraction
EM	Sr/Y logtest = logical test to set to 0 the negative Sr/Y values
EN	Logtest Psaturation = logical test to exclude illogical simulations which correspond to pressure of saturation values higher than the pressure of accumulation and at the same time being characterized by excess H_2O at the pressure of accumulation

Table S3.2. Description of all parameters used in simulations for melt injection rate of 50 mm/a through a disk of 10000 m radius (equivalent to 0.016 km^3/a) (Supplementary Dataset 2).

A	Time = time since first injection starts (ka): random between 51526 and 200000 a
B	H_2O parent = wt.% content of H_2O in parent magma (random between 2 and 4 wt.%)
C	H_2O assimilant = wt.% content of H_2O in assimilant (random between 0.5 and 1 wt.%)
D	P (kbars) = pressure at which magma accumulation occurs: linear function of time according to the underplating of magma at the fixed flux rate of 50 mm/a: =0.0000075*time+1.50015
E	SiO_2 total = SiO_2 relationship to melt fraction based on the empirical equation SiO_2=35.43629*F^2-68.8591*F+82.43897, where F = melt fraction
F	Melt fraction = fraction of melt (=AG: see below)
G	a Pressure = a parameter of the second order equation (y=ax^2+bx +c) describing the total melt fraction as a function of P
H	b Pressure = b parameter of the second order equation (y=ax^2+bx +c) describing the total melt fraction as a function of P
I	c Pressure = c parameter of the second order equation (y=ax^2+bx +c) describing the total melt fraction as a function of P
J	P saturation = pressure at which magma is H_2O-saturated as a function of the second order polynomial equation P saturation = [a Pressure]*[H_2O in melt]^2 + [b Pressure]*[H2O in melt] + [c Pressure]
K	Delta P = P of magma formation minus pressure of H_2O saturation for that magma
L	H_2O % in melt = wt.% content of soluble H_2O in the melt
M	Volume mobile magma km^3 = volume of mobile magma (melt fraction >60%) parameterized from Annen (2009)[2] (=0.00657*time-338.52065)
N	% mobile melt = random value between 60 and 80%
O	Volume melt (km^3) = effective volume of 100% melt in km^3
P	Volume melt (m^3) = effective volume of 100% melt in m^3
Q	Mass melt (tons) = mass of melt for a density of 2.5 g/cm^3 (dacite)
R	Volume magma injected (km^3) = volume of injected magma at specific times parameterized from Annen (2009)[2] (=0.01566*time+31.01613)
S	fixed rate (km^3/a) =fixed magma flux of 0.016 km^3/a
T	Volume magma injected (m^3) = W in m^3
U	Mass magma injected (tons) = volume magma injected multipplied by the density of magma (2.5)
V	% mass of melt = % of mass of melt with respect to the injected mass
W	% mass of melt = V
X	Tons melt = [Cell P below]*2.5
Y	Melt % = W
Z	Total melt % = V
AA	H_2O wt% in residual melt = H_2O wt.% in residual melt calculated for 30% residual melt according to: Residual H_2O=100*(H_2O parent)*30^{-1}
AB	log test H_2O wt% in residual melt =100*(H_2O parent)*(residual melt fraction)$^{-1}$
AC	a H_2O = a parameter of the second order equation (y=ax^2+bx +c) describing the dependence on pressure of the H_2O content of a silicate melt of a random composition (=melt fraction) paramaterized from the P-X dependency of H_2O solubility of VolatalieCalc (Newton and Lowenstern, 2002)[3].
AD	b H_2O = b parameter of the second order equation (y=ax^2+bx +c) describing the dependence on pressure of the H_2O content of a silicate melt of a random composition (=melt fraction) paramaterized from the P-X dependency of H_2O solubility of VolatalieCalc (Newton and Lowenstern, 2002)[3].
AE	c H_2O = c parameter of the second order equation (y=ax^2+bx +c) describing the dependence on pressure of the H_2O content of a silicate melt of a random composition (=melt fraction) paramaterized from the P-X dependency of H_2O solubility of VolatalieCalc (Newton and Lowenstern, 2002)[3].
AF	dacite = % residual melt fraction for a dacite (fixed at 27%)
AG	residual melt Annen logical test for H_2O solubility (F=100-10%) = AK*S
AH	H_2O solubility in melt (F =100-10 %) = H_2O solubility in the residual melt at the random

	pressure and residual melt fraction (logical test corrected) of the same raw
AI	log test H_2O solub = AH
AJ	H_2O wt% in melt logical test 100-10% F range = H_2O wt% in residual melt logical test 100-10% F range = effective H_2O wt% content of the residual melt corrected for oversaturation (i.e., if the theoretical amount of H_2O in the residual melt is higher than the maximum permissible H_2O content in that melt composition and at that specific P, then the latter value is taken as the effective H_2O content of that simulation and the remainder H_2O is in excess and must have been exsolved).
AK	Excess H_2O % = log test H_2O wt% in residual melt - log test H_2O solubility
AL	H_2O residual (tons) = tons of H_2O in hydrou smelt
AM	Excess H_2O (tons) = tons of exsolved H_2O from saturated melts
AN	log test excess H_2O (tons) = excess H_2O (tons) corrected to exclude spurious values from undersaturated melts
AO	new total hydrous melt = tons of hydrous melt
AP	H_2O in residual melt (tons) = AQ
AQ	Excess H_2O total in melt (tons) = AR
AR	total melt ton = V
AS	% H_2O excess = AP
AT	excess H_2O tons = tons of excess H_2O
AU	kD = KD fluid-melt for Cu with random range between 2 and 100
AV	Cu total melt ppm = BH
AW	Cu tot tons = tons of total Cu in magma
AX	Cu res melt ppm = Cu ppm in the residual magma after fluid exsolution
AY	Cu excess fluid ppm = Cuu ppm in exsolved fluid
AZ	Cu tot res melt Mt = Mt of Cu in residual melt after fluid exsolution
BA	Cu tot in excess fluid Mt = Mt of Cu in exsolved fluid
BB	Cu tot Mt = AZ + BA
BC	melt tons = AO
BD	a Cu = a parameter of the second order equation ($y=ax^2+bx+c$, where y = Cu (ppm) and x = SiO_2 (wt.%): Fig. 1 below) describing the Cu concentration variability at any SiO_2 value parameterized from the median values of Cu and SiO_2 from continental arc rocks (from Chiaradia, 2014)[4]
BE	b Cu = b parameter of the second order equation ($y=ax^2+bx+c$, where y = Cu (ppm) and x = SiO_2 (wt.%): Fig. 1 below) describing the Cu concentration variability at any SiO_2 value parameterized from the median values of Cu and SiO_2 from continental arc rocks (from Chiaradia, 2014)[4]
BF	c Cu = c parameter of the second order equation ($y=ax^2+bx+c$, where y = Cu (ppm) and x = SiO_2 (wt.%): Fig. 1 below) describing the Cu concentration variability at any SiO_2 value parameterized from the median values of Cu and SiO_2 from continental arc rocks (from Chiaradia, 2014)[4]
BG	Cu = Cu ppm in 2nd residual melt after complete H_2O exsolution
BH	Cu ppm melt = BG
BI	Cu Mt tons in magma = Cu Mt in 2nd residual melt after complete H_2O exsolution
BJ	M res hydrous melt ton = AO
BK	% H_2O in hydrous melt = AB
BL	exsolvable H_2O ton = tons of exsolvable H_2O in hydrous melt
BM	KD Cu = AU
BN	Cu total melt ppm = AV
BO	Cu tot ton = tons of Cu in residual melt
BP	Cu res melt = Cu ppm in residual melt
BQ	Cu fluid = Cu ppm in exsolvable fluid
BR	Cu tot res melt Mt = Mt of Cu in residual melt
BS	Cu tot in exsolvable fluid Mt = Mt of Cu in exsolvable fluid
BT	Cu tot Mt = Cu Mt total (sum of BW and BX)
BU	50% Cu fluid Mt = 50% of exsolvable Cu
BV	Molar V H_2O = molar volume of H_2O at the random pressure of the corresponding raw
BW	H_2O% in hydrous melt= wt% of H_2O in over or undersaturated hydrous melt

BX	Moles of excess H_2O = moles of H_2O exsolved from oversaturated melts
BY	Volume cc excess H_2O = cubic centimeters of H_2O exsolved from oversaturated melts
BZ	Volume km^3 excess H_2O = volume (km^3) of H_2O exsolved from oversaturated melts
CA	Volume km^3 total melt = volume (km^3) of total melt
CB	Volume % excess H_2O = volume % of H_2O exsolved from oversaturated melts
CC	Volume % H_2O logical test = logical test for Volume % excess H_2O (if >0 then = excess H_2O, if not = 0)
CD	Flux of Cu transported by excess fluid (t/a) = amount of Cu (tons) contained in excess fluid liberated at any time of the magma evolution divided by the time elapsed since onset of magmatic evolution
CE	aa- = minimum value of the "aa parameter" describing the second order polynomial pressure dependency of the "aa parameter" (=a eq below; a eq = aa*pressure^2 + ab*pressure + ac) in the third order polynomial equation describing the variation of Sr/Y values with melt fraction .
CF	aa+ = maximum value of the "aa parameter" describing the second order polynomial pressure dependency of the "a parameter" (=a eq below; a eq = aa*pressure^2 + ab*pressure + ac) in the third order polynomial equation describing the variation of Sr/Y values with melt fraction .
CG	aa = random value of the "aa parameter" between minimum and maximum values above describing the pressure dependency of the "a parameter" (=a eq below; a eq = aa*pressure^2 + ab*pressure + ac) in the third order polynomial equation describing the variation of Sr/Y values with melt fraction .
CH	ab- = minimum value of the "ab parameter" describing the second order polynomial pressure dependency of the "a parameter" (=ab eq below; a eq = aa*pressure^2 + ab*pressure + ac) in the third order polynomial equation describing the variation of Sr/Y values with melt fraction .
CI	ab+ = maximum value of the "ab parameter" describing the second order polynomial pressure dependency of the "a parameter" (=a eq below; a eq = aa*pressure^2 + ab*pressure + ac) in the third order polynomial equation describing the variation of Sr/Y values with melt fraction .
CJ	ab = random value of the "ab parameter" between minimum and maximum values above describing the pressure dependency of the "a parameter" (=a eq below; a eq = aa*pressure^2 + ab*pressure + ac) in the third order polynomial equation describing the variation of Sr/Y values with melt fraction .
CK	ac- = minimum value of the "ac parameter" describing the second order polynomial pressure dependency of the "a parameter" (=a eq below; a eq = aa*pressure^2 + ab*pressure + ac) in the third order polynomial equation describing the variation of Sr/Y values with melt fraction.
CL	ac+ = maximum value of the "ac parameter" describing the second order polynomial pressure dependency of the "a parameter" (=a eq below; a eq = aa*pressure^2 + ab*pressure + ac) in the third order polynomial equation describing the variation of Sr/Y values with melt fraction .
CM	ac = random value of the "ac parameter" between minimum and maximum values above describing the pressure dependency of the "a parameter" (=a eq below; a eq = aa*pressure^2 + ab*pressure + ac) in the third order polynomial equation describing the variation of Sr/Y values with melt fraction .
CN	a eq = aa*pressure^2 + ab*pressure + ac; a eq is the pressure-dependent variation of the a parameter in the third order polynomial equation (Sr/Y = [a eq]*[melt fraction]^3 + [b eq]*[melt fraction]^2 + [c eq]*[melt fraction] + [d eq]) describing the variation of Sr/Y values with melt fraction .
CO	ba- = minimum value of the "ba parameter" describing the second order polynomial pressure dependency of the "b parameter" (=b eq below; b eq = ba*pressure^2 + bb*pressure + bc) in the third order polynomial equation describing the variation of Sr/Y values with melt fraction .
CP	ba+ = maximum value of the "ba parameter" describing the second order polynomial pressure dependency of the "b parameter" (=b eq below; b eq = ba*pressure^2 + bb*pressure + bc) in the third order polynomial equation describing the variation of Sr/Y values with melt fraction .

CQ	ba = random value of the "ba parameter" between minimum and maximum values above describing the pressure dependency of the "b parameter" (=b eq below; b eq = ba*pressure^2 + bb*pressure + bc) in the third order polynomial equation describing the variation of Sr/Y values with melt fraction .
CR	bb- = minimum value of the "bb parameter" describing the second order polynomial pressure dependency of the "b parameter" (=b eq below; b eq = ba*pressure^2 + bb*pressure + bc) in the third order polynomial equation describing the variation of Sr/Y values with melt fraction.
CS	bb+ = maximum value of the "bb parameter" describing the second order polynomial pressure dependency of the "b parameter" (=c eq below; b eq = aa*pressure^2 + ab*pressure + ac) in the third order polynomial equation describing the variation of Sr/Y values with melt fraction .
CT	bb = random value of the "bb parameter" between minimum and maximum values above describing the pressure dependency of the "b parameter" (=b eq below; b eq = aa*pressure^2 + ab*pressure + ac) in the third order polynomial equation describing the variation of Sr/Y values with melt fraction .
CU	bc- = minimum value of the "bc parameter" describing the second order polynomial pressure dependency of the "b parameter" (=b eq below; b eq = aa*pressure^2 + ab*pressure + ac) in the third order polynomial equation describing the variation of Sr/Y values with melt fraction .
CV	bc+ = maximum value of the "bc parameter" describing the second order polynomial pressure dependency of the "b parameter" (=b eq below; b eq = aa*pressure^2 + ab*pressure + ac) in the third order polynomial equation describing the variation of Sr/Y values with melt fraction .
CW	bc = random value of the "bc parameter" between minimum and maximum values above describing the pressure dependency of the "b parameter" (=b eq below; b eq = aa*pressure^2 + ab*pressure + ac) in the third order polynomial equation describing the variation of Sr/Y values with melt fraction .
CX	b eq = ba*pressure^2 + bb*pressure + bc; b eq is the pressure-dependent variation of the b parameter in the third order polynomial equation (Sr/Y = [a eq]*[melt fraction]^3 + [b eq]*[melt fraction]^2 + [c eq]*[melt fraction] + [d eq]) describing the variation of Sr/Y values with melt fraction .
CY	ca- = minimum value of the "ca parameter" describing the second order polynomial pressure dependency of the "c parameter" (=c eq below; c eq = ca*pressure^2 + cb*pressure + cc) in the third order polynomial equation describing the variation of Sr/Y values with melt fraction (**s see above**).
CZ	ca+ = maximum value of the "ca parameter" describing the second order polynomial pressure dependency of the "c parameter" (=c eq below; c eq = ca*pressure^2 + cb*pressure + cc) in the third order polynomial equation describing the variation of Sr/Y values with melt fraction .
DA	ca = random value of the "ca parameter" between minimum and maximum values above describing the pressure dependency of the "c parameter" (=c eq below; c eq = ca*pressure^2 + cb*pressure + cc) in the third order polynomial equation describing the variation of Sr/Y values with melt fraction .
DB	cb- = minimum value of the "cb parameter" describing the second order polynomial pressure dependency of the "c parameter" (=c eq below; c eq = ca*pressure^2 + cb*pressure + cc) in the third order polynomial equation describing the variation of Sr/Y values with melt fraction .
DC	cb+ = maximum value of the "cb parameter" describing the second order polynomial pressure dependency of the "c parameter" (=c eq below; c eq = ca*pressure^2 + cb*pressure + cc) in the third order polynomial equation describing the variation of Sr/Y values with melt fraction .
DD	cb = random value of the "cb parameter" between minimum and maximum values above describing the pressure dependency of the "c parameter" (=c eq below; c eq = ca*pressure^2 + cb*pressure + cc) in the third order polynomial equation describing the variation of Sr/Y values with melt fraction .
DE	cc- = minimum value of the "cc parameter" describing the second order polynomial pressure dependency of the "c parameter" (=c eq below; c eq = ca*pressure^2 + cb*pressure + cc) in the third order polynomial equation describing the variation of

	Sr/Y values with melt fraction .
DF	cc+ = maximum value of the "cc parameter" describing the second order polynomial pressure dependency of the "c parameter" (=c eq below; c eq = ca*pressure^2 + cb*pressure + cc) in the third order polynomial equation describing the variation of Sr/Y values with melt fraction .
DG	cc = random value of the "cc parameter" between minimum and maximum values above describing the pressure dependency of the "c parameter" (=c eq below; c eq = ca*pressure^2 + cb*pressure + cc) in the third order polynomial equation describing the variation of Sr/Y values with melt fraction .
DH	c eq = ca*pressure^2 + cb*pressure + cc; c eq is the pressure-dependent variation of the a parameter in the third order polynomial equation (Sr/Y = [a eq]*[melt fraction]^3 + [b eq]*[melt fraction]^2 + [c eq]*[melt fraction] + [d eq]) describing the variation of Sr/Y values with melt fraction .
DI	da- = minimum value of the "da parameter" describing the second order polynomial pressure dependency of the "d parameter" (=d eq below; d eq = da*pressure^2 + db*pressure + dc) in the third order polynomial equation describing the variation of Sr/Y values with melt fraction .
DJ	da+ = maximum value of the "da parameter" describing the second order polynomial pressure dependency of the "cd parameter" (=d eq below; d eq = da*pressure^2 + db*pressure + dc) in the third order polynomial equation describing the variation of Sr/Y values with melt fraction .
DK	da = random value of the "da parameter" between minimum and maximum values above describing the pressure dependency of the "c parameter" (=d eq below; d eq = da*pressure^2 + db*pressure + dc) in the third order polynomial equation describing the variation of Sr/Y values with melt fraction .
DL	db- = minimum value of the "db parameter" describing the second order polynomial pressure dependency of the "d parameter" (=d eq below; d eq = da*pressure^2 + db*pressure + dc) in the third order polynomial equation describing the variation of Sr/Y values with melt fraction .
DM	db+ = maximum value of the "db parameter" describing the second order polynomial pressure dependency of the "d parameter" (=d eq below; d eq = da*pressure^2 + db*pressure + dc) in the third order polynomial equation describing the variation of Sr/Y values with melt fraction .
DN	db = random value of the "db parameter" between minimum and maximum values above describing the pressure dependency of the "d parameter" (=d eq below; d eq = da*pressure^2 + db*pressure + dc) in the third order polynomial equation describing the variation of Sr/Y values with melt fraction .
DO	dc- = minimum value of the "dc parameter" describing the second order polynomial pressure dependency of the "d parameter" (=d eq below; d eq = da*pressure^2 + db*pressure + dc) in the third order polynomial equation describing the variation of Sr/Y values with melt fraction .
DP	dc+ = maximum value of the "dc parameter" describing the second order polynomial pressure dependency of the "d parameter" (=d eq below; d eq = da*pressure^2 + cdb*pressure + dc) in the third order polynomial equation describing the variation of Sr/Y values with melt fraction .
DQ	dc = random value of the "dc parameter" between minimum and maximum values above describing the pressure dependency of the "d parameter" (=d eq below; d eq = da*pressure^2 + db*pressure + dc) in the third order polynomial equation describing the variation of Sr/Y values with melt fraction .
DR	d eq = da*pressure^2 + db*pressure + dc; d eq is the pressure-dependent variation of the a parameter in the third order polynomial equation (Sr/Y = [a eq]*[melt fraction]^3 + [b eq]*[melt fraction]^2 + [c eq]*[melt fraction] + [d eq]) describing the variation of Sr/Y values with melt fraction .
DS	Sr/Y = [a eq]*[melt fraction]^3 + [b eq]*[melt fraction]^2 + [c eq]*[melt fraction] + [d eq]) describes the variation of Sr/Y values with melt fraction .
DT	Sr/Y logtest = logical test to set to 0 the negative Sr/Y values

References

[1] Annen, C., Blundy, J. D., & Sparks, R. S. J. The genesis of intermediate and silicic magmas in deep crustal hot zones. *J. Pet.* **47**, 505–539 (2006).
[2] Annen, C. From plutons to magma chambers: Thermal constraints on the accumulation of eruptible silicic magma in the upper crust. *Earth Planet. Sci. Lett.* **284**, 409–416 (2009).
[3] Newman, S. & Lowenstern, J. B. VolatileCalc: a silicate melt-H_2O-CO_2 solution model written in Visual Basic for Excel. *Computers and Geosciences* **28**, 597-604 (2002).
[4] Chiaradia M., Copper enrichment in arc magmas controlled by over- riding plate thickness. *Nature Geoscience* **7**, 43–46 (2014).
