# Supplementary material for: Stochastic modelling of deep magmatic controls on porphyry copper deposit endowment
Source: Sci Rep. 2017 Mar 15;7:44523. doi: 10.1038/srep44523 (PMC5353633; doi:10.1038/srep44523)
Supplement: Supplementary Information 4 [file srep44523-s4.pdf]

Supplementary Information 4

Figure S4.1

Stochastic modelling of deep magmatic controls on porphyry copper deposit endowment

Massimo Chiaradia*, Luca Caricchi

Department of Earth Sciences, University of Geneva, Rue des Maraîchers 13, 1205 Geneva, Switzerland

**Corresponding author: Tel.: +41 22 379 66 34; Fax: +41 22 379 32 10; e-mail: Massimo.Chiaradia@unige.ch*

Figure S4.1: Histogram of Monte Carlo simulations of H_2O concentrations for magmatic systems generated under an average arc magma flux of 0.0009 km^3/a, capable of releasing >30 Mt Cu. The greatest majority (83%) of these most productive magmatic systems are characterized by H_2O contents between 6 and 12 wt.% (see also Ref. 1).

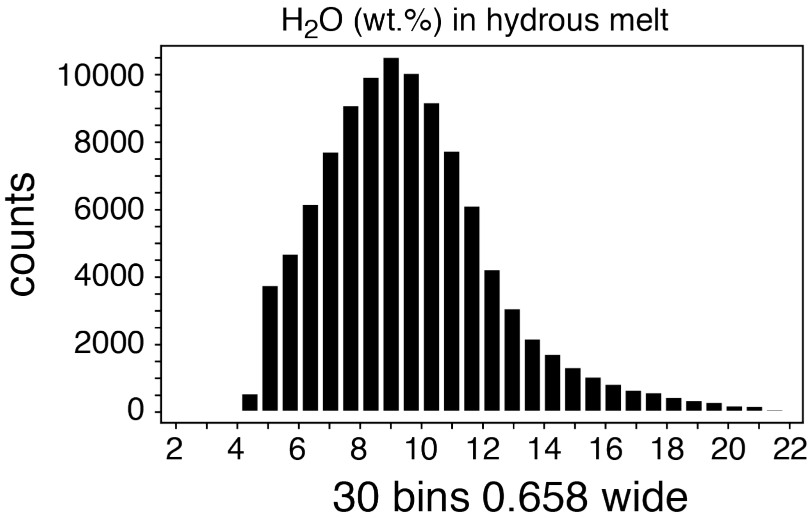

References

[1] Lu, Y.-J. *et al.*, Fluid flux melting generated post-collisional high Sr/Y copper ore-forming water-rich magmas in Tibet. *Geology* **43**, 583-586 (2015).
